# Identification of Disease-Associated Cryptococcal Proteins Reactive With Serum IgG From Cryptococcal Meningitis Patients

**DOI:** 10.3389/fimmu.2021.709695

**Published:** 2021-07-23

**Authors:** A. Elisabeth Gressler, Daniela Volke, Carolina Firacative, Christiane L. Schnabel, Uwe Müller, Andor Krizsan, Bianca Schulze-Richter, Matthias Brock, Frank Brombacher, Patricia Escandón, Ralf Hoffmann, Gottfried Alber

**Affiliations:** ^1^ Institute of Immunology, Faculty of Veterinary Medicine, Leipzig University, Leipzig, Germany; ^2^ Institute of Bioanalytical Chemistry, Leipzig University, Leipzig, Germany; ^3^ Studies in Translational Microbiology and Emerging Diseases (MICROS) Research Group, School of Medicine and Health Sciences, Universidad del Rosario, Bogota, Colombia; ^4^ Fungal Genetics and Biology Group, School of Life Sciences, University of Nottingham, Nottingham, United Kingdom; ^5^ International Centre for Genetic Engineering and Biotechnology (ICGEB), Cape Town Component, Cape Town, South Africa; ^6^ Microbiology Group, Instituto Nacional de Salud, Bogota, Colombia

**Keywords:** *Cryptococcus neoformans*, immunoproteomics, cryptococcal meningitis, humoral immunity, human samples, fungal infection

## Abstract

*Cryptococcus neoformans*, an opportunistic fungal pathogen ubiquitously present in the environment, causes cryptococcal meningitis (CM) mainly in immunocompromised patients, such as AIDS patients. We aimed to identify disease-associated cryptococcal protein antigens targeted by the human humoral immune response. Therefore, we used sera from Colombian CM patients, with or without HIV infection, and from healthy individuals living in the same region. Serological analysis revealed increased titers of anti-cryptococcal IgG in HIV-negative CM patients, but not HIV-positive CM patients, compared to healthy controls. In contrast, titers of anti-cryptococcal IgM were not affected by CM. Furthermore, we detected pre-existing IgG and IgM antibodies even in sera from healthy individuals. The observed induction of anti-cryptococcal IgG but not IgM during CM was supported by analysis of sera from *C. neoformans*-infected mice. Stronger increase in IgG was found in wild type mice with high lung fungal burden compared to IL-4Rα-deficient mice showing low lung fungal burden. To identify the proteins targeted by human anti-cryptococcal IgG antibodies, we applied a quantitative 2D immunoproteome approach identifying cryptococcal protein spots preferentially recognized by sera from CM patients or healthy individuals followed by mass spectrometry analysis. Twenty-three cryptococcal proteins were recombinantly expressed and confirmed to be immunoreactive with human sera. Fourteen of them were newly described as immunoreactive proteins. Twelve proteins were classified as disease-associated antigens, based on significantly stronger immunoreactivity with sera from CM patients compared to healthy individuals. The proteins identified in our screen significantly expand the pool of cryptococcal proteins with potential for (i) development of novel anti-cryptococcal agents based on implications in cryptococcal virulence or survival, or (ii) development of an anti-cryptococcal vaccine, as several candidates lack homology to human proteins and are localized extracellularly. Furthermore, this study defines pre-existing anti-cryptococcal immunoreactivity in healthy individuals at a molecular level, identifying target antigens recognized by sera from healthy control persons.

## Introduction

*Cryptococcus neoformans*, an encapsulated opportunistic fungal pathogen, is the main agent causing cryptococcosis, a fatal systemic disease (1). Cryptococcal infection occurs through inhalation of spores ubiquitously present in birds’ droppings (2–4), house dust (5) and decaying wood (6). However, the most common clinical manifestation of cryptococcosis is not pulmonary, but disseminated disease manifesting mostly as cryptococcal meningitis (CM) (7).

The main risk factor for development of systemic cryptococcal disease is impaired cell-mediated immunity (8), typically occurring in HIV-positive patients with AIDS. These patients account for 80-95% of all cases (7, 8), but individuals receiving immunosuppressive drugs are also at risk (8). Cases of CM have also been reported in immunocompetent persons (9), and in persons with increased M2 polarization of brain macrophages (10). Additionally, several case reports describe CM or other forms of disseminated cryptococcal disease in persons with humoral immunity defects like immunoglobulin (Ig)G-deficiencies (11–14) or X-linked hyper-IgM syndrome, which is characterized by reduced IgG and IgA serum levels and normal or elevated serum IgM (15–18). CM has also been found in patients with reduced percentage of IgM-producing memory B cells (19), indicating the contribution of humoral immunity in anti-cryptococcal defense for control of *C. neoformans*.

Although both, absolute case number and mortality rates of CM, decreased in recent years due to facilitated diagnosis through serum cryptococcal antigen screening and treatment of underlying HIV infection with anti-retroviral therapy, CM remains a severe health issue especially in low-income and middle-income countries, causing an estimated number of 181,000 deaths per year in the world (20, 21). Additionally, access to antifungals is limited in most low-income countries and when available, antifungal agents cause severe side-effects (22). Therefore, the treatment of fungal disease using immunotherapeutic approaches or the design of anti-fungal vaccines is gaining increased attention (23–26).

Cell-mediated immunity seems critical for cryptococcal clearance (27, 28). Nevertheless, several studies indicate importance of humoral immunity for protection from infection (29, 30). In addition to defects in B cells and antibody-mediated immunity constituting a risk factor for cryptococcal disease in humans (11–17), mice lacking B-1a B cells (31, 32), or soluble IgM antibodies (33) showed significantly higher fungal burden and decreased survival upon cryptococcal challenge. Anti-cryptococcal antibodies produced by humans (34, 35) and mice (36–39) were shown to act as opsonins and promote phagocytosis and killing of cryptococcal cells *in vitro*. Consequently, several studies using cryptococcal antigenic compounds, with or without additional adjuvants, for vaccination of mice or rats elicited an antibody-mediated response protective against subsequent cryptococcal challenge (24, 40–46). Therefore, targeting the antibody-mediated response appears as a promising approach for prevention and treatment of CM.

Various studies demonstrated the ubiquitous presence of antibodies in human sera directed against cryptococcal capsular polysaccharides (47–56), mannoproteins (57), and cryptococcal proteins (58–62), regardless of predisposing HIV infection or even previous history of cryptococcal disease. However, the *C. neoformans* proteins targeted by these human antibodies have not been identified so far. Previous studies focused on identification of proteins that are immunoreactive with sera from mice (45, 63, 64), and koalas (65), or immunoreactive proteins from *Cryptococcus gattii* targeted by murine (46) or human antibodies (66). Those studies identified proteins contained in immunoreactive cryptococcal protein spots, but did not confirm immunoreactivity of the identified proteins by subsequent recombinant expression.

Therefore, we aimed to identify immunoreactive *C. neoformans* proteins using human sera from Colombian CM patients with or without underlying HIV infection and sera from healthy individuals (controls) living in Colombia to guarantee similar environmental exposure to *C. neoformans*. We were able to define IgG as the predominant isotype mounted in a disease-specific manner against *C. neoformans*. Using a quantitative immunoproteomic approach based on 2-dimensional gel electrophoresis and subsequent recombinant expression, we identified disease-associated cryptococcal proteins, defined by significantly stronger reactivity with sera from CM patients compared to healthy individuals. Our study therefore critically expands the pool of immunoreactive cryptococcal protein antigens associated with CM. Some of these proteins are promising targets for (i) anti-cryptococcal chemotherapy or (ii) development of an anti-cryptococcal, or even pan-fungal, vaccine.

## Material and Methods

### Patients and Sera

Sera were obtained from HIV-positive (CD4^+^ T cells <250 cells/µL) and HIV-negative Colombian cryptococcal meningitis (CM) patients at the time of diagnosis. Diagnosis was secured by positive culture from cerebrospinal fluid for *C. neoformans* and visualization of the encapsulated yeasts, stained with Indian ink, by direct microscopy. Detection of cryptococcal antigen (CrAg) in serum samples or cerebrospinal fluid by latex agglutination system (CALAS^®^) was carried out after diagnosis. The following clinical data was collected: age, sex, HIV status, CD4 count (for HIV-positive patients). Apart from the immunosuppression with HIV, other risk factors were also noted as underlying conditions. Sera from healthy Colombian individuals without cryptococcosis or any other mycosis were used as controls. Serum samples were collected in hospitals from different states of Colombia and sent to the Microbiology Group of the National Institute of Health, Bogota, Colombia, as part of the surveillance program for cryptococcosis. These samples belong to the sera collection of the Microbiology Group and were collected between 1990 and 2014. Informed consent was obtained from the patients prior to investigation. Human samples were used with approval from the Technical Committee of Research (CTIN) and the Ethical Committee for Research (CEIN) of the National Institute of Health, Bogota, Colombia, Memorandum No 3000-12829 of 2015. Sera from healthy controls were obtained with the approval of the ethical committee of Corporación para Investigaciones Biológicas (CIB) and Hospital La Maria IRB Number 7250 in Medellin, Colombia. An informed consent form was signed by all people enrolled in the study. All clinical information from the participants in the study was anonymized.

### Mouse Experiments

Wild type (WT) and IL-4Rα-deficient (IL-4Rα^-/-^) Balb/cJ (67) adult female mice were infected intranasally with 500 colony forming units (CFU) of *C. neoformans* strain 1841 (serotype D) yeasts. Cryptococcal cells were prepared before infection as previously described (68). For determination of fungal burden in the lung, mice were sacrificed at different time points, lungs were homogenized and plated on Sabouraud dextrose agar. Colonies were counted after two days of incubation at 30°C and number of CFU per lung were calculated. Serum samples were obtained at the endpoint of the experiment for measurement of anti-cryptococcal Ig titers. The mice were maintained under specific pathogen-free conditions, according to the guidelines authorized by the Animal Care and Usage Committee of the “Landesdirektion Sachsen” (www.lds.sachsen.de, Chemnitz, Germany) with food and water *ad libitum*. All infection experiments were carried out in accordance with the guidelines of the Animal Care and Use Committee of the “Landesdirektion Sachsen” according to the approved protocols with numbers 24-9168.11-15/05 and 24-9168 TVV 16/09.

### Flow Cytometry Measurement of *C. neoformans*-Specific Immunoglobulins

*C. neoformans* serotype A strain H99 cells were recovered from 10% skim milk stocks stored at -80°C and washed once with phosphate buffered saline (PBS). From cell suspension, 2x10^5^ (human IgG analysis) or 5x10^5^ (human IgM analysis, murine IgM and IgG analysis) cryptococcal cells were incubated with human serum samples diluted 1:10 in FACS buffer (3% FCS, 0.1% NaN_3_ in PBS) for 30 min at 4°C. Cells were washed once with FACS buffer and once with PBS and incubated with Fixable Viability Dye eFluor™ 780 (1:500 in PBS; ThermoFisher Scientific, Waltham, MA, USA) for 20 min at 4°C. Afterwards, cells were washed twice with FACS buffer and incubated for 30 min at 4°C with secondary antibodies from SouthernBiotech (Birmingham, AL, USA) labeled with FITC, diluted as indicated in FACS buffer: anti-human IgG-FITC (1:1,000; Cat. No. 2040-02), anti-human IgM-FITC (1:500; Cat. No. 2020-02), goat-anti mouse IgG-FITC, human adsorbed (1:500; Cat. No. 1030-02), or goat anti-mouse IgM-FITC (1:500; Cat. No. 1021-02). Thereafter, cells were washed three times with FACS buffer and 2% parafomaldehyde was added for fixation for 20 min at 4°C. Cells were washed once and resuspended in FACS buffer. Measurement of median fluorescent intensity of 10,000 events was performed using a BD LSRFortessa™ (Becton Dickinson, Franklin Lakes, NJ, USA). Analysis was carried out using FlowJo v10 (BD Life Sciences, Ashland, OR, USA). Gating strategy is shown in **Supplementary Figure 1A**.

For verification of *C. neoformans* specificity of the signal, human sera were incubated with 2x10^5^ (IgG)/5x10^5^ (IgM) *C. neoformans* H99 or *Candida albicans* SC5314 cells for 30 min at 4°C prior to detection of anti-cryptococcal antibodies. Sera were separated from the cells by centrifugation and pre-absorbed sera were transferred to *C. neoformans* H99 cells, followed by incubation for another 30 min at 4°C as described above. The resulting post-absorption values were calculated as percent of the MFI signal without pre-absorption to determine “quenchable sera”.

### Isolation of Cryptococcal Proteins

*C. neoformans* H99 (serotype A) and 1841 (serotype D) cells were recovered from 10% skim milk stocks stored at -80°C and grown independently for 48 h in Sabouraud dextrose broth while shaking (80 rpm) at 30°C. Cells were harvested by centrifugation and washed twice with 250 mM sucrose. The pellet was resuspended in lysis buffer [5 mM Tris/HCl pH 7.5, 2.5 mM EDTA, 0.5X protease inhibitor cocktail (Roche, Basel, Switzerland)] additionally containing 4% 3-[(3-cholamidopropyl) dimethylammonio]-1-propanesulfonate (CHAPS, Cat. No. 1479, Carl Roth, Karlsruhe, Germany) and 50 mM dithiothreitol (DTT, Cat. No. 6908, Carl Roth, Germany). The cell suspension was transferred into a mortar, frozen with liquid nitrogen and homogenized with a pestle twice. Afterwards, homogenates were centrifuged and supernatant was recovered. Protein content was estimated using Bradford reagent (Carl Roth, Karlsruhe, Germany). Proteins were precipitated with 10% trichloroacetic acid over night at -20°C and centrifuged. After removal of the supernatant, the pellet was washed three times with ice-cold acetone and air-dried. The protein pellet was dissolved in a solution containing 7 M urea, 2 M thiourea, and 4% CHAPS and protein content was estimated using Bradford reagent (Carl Roth, Germany).

### Isolation of Cryptococcal Capsular Polysaccharide

*C. neoformans* H99 and 1841 cells were recovered from 10% skim milk stocks stored at -80°C and grown independently for four days in Sabouraud dextrose broth while shaking (80 rpm) at 30°C. Cell supernatant was harvested by centrifugation and sodium acetate was added slowly up to a concentration of 10% w/v while stirring. Polysaccharide precipitation was performed by addition of 2.5 volumes of 99.5% ethanol and incubation for three days at room temperature (RT). After removal of the supernatant by centrifugation, polysaccharides were air dried and dissolved in deionized H_2_O. Polysaccharide concentration was measured by the method of Dubois (69).

### ELISA Analysis of Human and Murine Samples

Concentration of IgG and IgM antibodies in human and murine sera were determined by ELISA analysis. Briefly, 96 well round bottom plates were coated overnight at 4°C with the following antibodies from SouthernBiotech (AL, USA) diluted in carbonate buffer: goat anti-human IgG, mouse adsorbed (0.5 µg/mL, Cat. No. 2044-01), goat anti-human IgM (1 µg/mL, Cat. No. 2020-01), goat anti-mouse IgG, human adsorbed (2.5 µg/mL, Cat. No. 1030-01), goat anti-mouse IgM (2.5 µg/mL, Cat. No. 1021-01). The plates were washed once with PBS containing 0.05% Tween-20 (PBS-T) and blocked with PBS containing 0.5% BSA and 0.1% gelatin (RT, 1.5 h). The following Igs from SouthernBiotech (AL, USA) were used as standards: Human IgG (starting dilution 0.25 µg/mL, Cat. No. 0150-01), human IgM (starting dilution 1 µg/mL, Cat. No. 0158L-01), mouse IgG (starting dilution 0.5 µg/mL, Cat. No. 0107-01) and mouse IgM (starting dilution 1 µg/mL, Cat. No. 0101-01). Sera were diluted in blocking buffer containing 0.05% Tween-20 and incubated for 2 h at RT, followed by washing with PBS-T. Afterwards, plates were incubated with HRP-labelled secondary antibodies from SouthernBiotech (AL, USA) specific for human IgG (Cat. No. 2040-05), human IgM (Cat. No. 2020-05), mouse IgG (Cat. No. 1030-05) or mouse IgM (Cat. No. 1021-05) diluted 1:5000, or 1:4000 for quantification of human Igs or murine Igs, respectively. After 2 h incubation, the plates were washed four times with PBST and developed with 3,3′,5,5′-tetramethylbenzidine (Kirkegaard & Perry Lab Inc (KPL), Gaithersburg, MD, USA). Colorimetric reaction was stopped when OD_650/480_ of the first standard dilution reached a value of 1.3 with 1 M H_3_PO_4_ and OD_450/630_ was measured using the SpectraMAx 340PC Photometer and SoftMaxPro (v5.0) software (Molecular Devices, San José, CA; USA) was used for calculation of immunoglobulin concentration.

For determination of *C. neoformans*-specific antibody titers, 96 well plates were coated over night at 4°C with proteins or polysaccharides (10 µg/mL in carbonate buffer), respectively. Plates were washed once with PBS-T and blocked with 5% skim milk dissolved in PBS for one hour at RT. Serum samples were diluted 1:400 up to 1:409,600 (IgG) or 1:100 up to 1:102400 (IgM) in serum diluent (5% skim milk dissolved in PBS containing 0.1% Tween-20) for human sera samples or 1:100 up to 1:102,400 (IgG and IgM) for analysis of mouse sera and incubated for two hours at RT. Next, plates were washed five times with PBS-T and incubated with secondary antibody from SouthernBiotech (Birmingham, AL, USA), diluted 1:5,000 in serum diluent (goat anti-human IgG-HRP (Cat. No. 2040-05); goat anti-human IgM-HRP (Cat. No. 2020-05); goat anti-mouse IgG-HRP (Cat. No. 1030-05), goat anti-mouse IgM-HRP, (Cat. No. 1021-05) for 2 hours at RT. Plates were washed five times with PBS-T and developed using 3,3′,5,5′-tetramethylbenzidine (KPL, MD, USA). Reaction was stopped by adding 1 M H_3_PO_4_ after 15 min (IgG, human samples), 20 min (IgM, human samples) or 30 min (IgG and IgM, murine samples) for detection. OD_450/630_ was measured using SpectraMAx 340PC Photometer and SoftMaxPro (v5.0) software (Molecular Devices, CA; USA). The titer of *C. neoformans*-reactive antibodies was defined as the highest dilution with an OD>0.1 after subtraction of serum background signal determined in wells without protein coating.

### Two-Dimensional (2D) Gel Electrophoresis and Immunoproteome Analysis

Protein solution containing *C. neoformans* H99 proteins purified by TCA precipitation was supplemented with DTT (50 mM), 1% BioLyte^®^ (BioRad, Hercules, CA, USA) and 0.001% bromophenol blue. SERVA IPG BlueStrips (3-10 NL, SERVA, Heidelberg, Germany) were rehydrated at RT with 100 µg of protein for six hours. Proteins were focused overnight using the PROTEAN IEF cell (BioRad, CA, USA) under the following conditions: active rehydration, 50 V for 6 h; Step 1, 150 V, rapid ramp for 1 h; Step 2, 300 V, rapid ramp for 1 h; Step 3, 1,000 V, linear ramp for 1 h; Step 4, 3,000 V, linear ramp for 2 h; Step 5, 3,000 V, rapid ramp for 2 h; and Step 6, 500 V for 12 h. Following isoelectric focusing, strips were soaked two times in equilibration solution (6 M urea, 2% sodium dodecyl sulfate (SDS), 50 mM Tris/HCl pH 8.8, 20% glycerol) and 1.) 2% DTT (Cat. No. 6908, Carl Roth, Germany) or 2.) 5% iodoacetamide (Cat. No. I6125, Sigma-Aldrich, St. Louis, MO, USA) for 15 min each. After equilibration, proteins were separated on acrylamide SDS gels containing 0.5% 2,2,2-trichloroethanol (TCE, Sigma-Aldrich, MO, USA) using the Owl™ Dual-Gel Vertical Electrophoresis Systems P8DS equipment (ThermoFisher Scientific, MA, USA). Proteins in the gels were stained with Coomassie Brilliant Blue G250 for cutting and digestion of protein spots. For detection of immunoreactive proteins fluorescent TCE staining was UV-activated for 1 min (ChemiDoc MP, BioRad, CA, USA) for protein visualization and proteins were transferred from the gel onto a nitrocellulose membrane by electroblotting in tank blots using the Mini Trans-Blot equipment (BioRad, CA, USA). After blotting, membranes were blocked with 1x BlueBlock PF blocking buffer for 1.5 h (Serva, Germany). Membranes were incubated with pooled human serum (4°C, overnight), diluted 1:1,000 in 1x BlueBlock PF. Membranes were washed three times with Tris-buffered saline (TBS), containing 0.05% Tween 20 (TBS-T) and incubated (RT, 1 h) with goat anti-human IgG-AlexaFluor^®^ 647 (SouthernBiotech Cat. No. 2040-31, AL, USA) diluted 1:2,500 in BlueBlock PF, followed by three washing steps with TBS-T. Total cryptococcal proteins stained with TCE were imaged by fluorescence in the stain free channel. Immunoreactive proteins were detected subsequently in the Cy5 channel using the ChemiDoc MP device (BioRad, CA, USA). Delta2D 4.8 software (DECODON, Greifswald, Germany) was used for quantification and analysis of immunoreactive protein spots, as well as mapping of the immunoreactive spots on the corresponding Coomassie-stained gel for mass spectrometry analysis. Analysis in Delta2D was carried out as follows: Spot boundaries were detected on a fused image, created from the protein spot patterns of all blots. Spots, detected at similar intensities among different experiments were considered in further analysis. Spot boundaries were transferred onto immunoblot signal images for quantification. Statistical analysis of immunoblot signals of serum sub-pools from HIV-positive CM patients, HIV-negative CM patients and healthy control patients was carried out using the T-test implemented in Delta2D relying on using the following parameters: Test design: between-subjects, used Welch approximation, alpha (overall threshold p-value): 0.01, p-values based on permutation, all permutations used: true, number of permutations per spot: 924, significance determined by standard Bonferroni correction, HCL: complete linkage, Euclidean Distance.

### In-Gel Digest and nRPC-ESI-MS/MS-TWIMS

Gel spots from the 2D gels and bands of the expressed proteins were excised with the ExQuest™ Spot Cutter (Bio-Rad Laboratories, Hercules, California, USA) and transferred into 0.5 mL reaction tubes. Gel pieces were washed three times (5 min, 100 µL 30% (v/v) acetonitrile in 50 mmol/L ammonium bicarbonate), dehydrated with acetonitrile (5 min, 100 µL), rehydrated with a mixture of 2 µL trypsin solution (Serva, Germany, 50 ng/µL in 3 mmol/L aqueous ammonium bicarbonate) and 18 µL of 3 mmol/l aqueous ammonium bicarbonate. After incubation (37°C, 4 h), supernatants were transferred to new 0.5 mL reaction tubes. Remaining gel pieces were washed once with 60% (v/v) aqueous acetonitrile containing 0.1% (v/v) formic acid and acetonitrile (20 µL per tube, RT, 5 min). Supernatants were transferred to the corresponding reaction tube and dried (60°C, 1 h) in a vacuum concentrator 5301 (Eppendorf Vertrieb Deutschland GmbH, Hamburg, Germany). The dried digests were dissolved in a mixture of 1.5 µL of acetonitrile containing 0.1% (v/v) formic acid (eluent B) and 48.5 µL of 0.1% aqueous formic acid (eluent A) and separated on a nanoACQUITY Ultra Performance LC™ (Waters Corp., Manchester, UK) system coupled online to a Q-TOF SYNAPT G2-Si instrument (Waters Corp., UK). Peptides were trapped on a nanoACQUITY Symmetry C18-column, internal diameter (ID) 180 µm, length 2 cm, particle diameter 5 µm, flow rate of 5 µL/min (3% eluent B, 6 min) on a C18-BEH 130 column (ID 75 µm, length 10 cm, particle diameter 1.7 µm; 35°C) at a flow rate of 0.3 µL/min using linear gradient from 3% to 40% eluent B in 18.5 min. The nanoESI source was equipped with a PicoTip Emmitter (New Objective, Littleton, US) at a spray voltage of 3 kV, sampling cone was 30 V, source offset 80 V, source temperature 100°C, cone gas flow 20 L/h, and nanoflow gas pressure 0.2 bar. Mass spectra were recorded in positive ion mode using a high-definition data-dependent acquisition approach (HD-DDA) for top 6 ions as described before (70). LC-MS/MS raw files were processed with the Mascot search engine (Version 2.7; Matrix Science Ldt., Waters, UK) using the following parameters Swissprot protein database (loaded 4^th^ November 2019), NCBI Cryptococcus (loaded on 21^th^ June 2018; 335 811 sequences), enzyme trypsin, 2 miss cleavage sides, as fixed modification cysteine carbamidomethylation (+57.022 Da), as variable modification methionine oxidation (+15.9949 Da), 20 ppm peptide tolerance and 0.08 Da fragment tolerance. Error tolerant was selected for MS/MS search. Proteins identified by at least three peptides and a protein score ≥50 were considered as confident.

### RNA Isolation and cDNA Synthesis

*C. neoformans* H99 (serotype A) and JEC21 (serotype D) cells were cultured in Sabouraud dextrose broth for 16 h at 30°C shaking at 80 rpm. Cells were washed once with PBS and transferred into a mortar and cells were frozen with liquid nitrogen and homogenized with a pestle twice. For the second freezing step, 500 µL NucleoZOL reagent (Macherey-Nagel, Düren, Germany) was added per 1x10^7^ cells. RNA extraction was performed according to the manufacturer’s protocol, except precipitation of RNA with isopropanol was performed for 2 h at -20°C. RNA was re-suspended in RNase-free water and concentration was determined using NanoDrop (ThermoScientific, MA, USA). cDNA synthesis was performed with High-Capacity cDNA Reverse Transcription Kit (Cat. No. 4368814, ThermoFisher, MA, USA) according to the manufacturer’s protocol, except only Oligo-dT primers were used.

### Recombinant Protein Expression

Genes of interest were amplified from *C. neoformans* H99 cDNA by polymerase chain reaction (PCR) using Phusion™ High-Fidelity DNA Polymerase (Cat. No. F530S, ThermoFisher, MA, USA). Primers are listed in **Supplementary Table 1**. PCR products and the target vector pET28a+ were purified using NucleoSpin^®^ PCR and gel clean-up kit (Cat. No. 740609, Macherey-Nagel, Germany) or the QIAprep^®^ Spin Miniprep Kit (Cat. No. 27106, Qiagen, Venlo, Netherlands), respectively. PCR products and the vector were digested for 16 h at 37°C in CutSmart buffer (New England Biolabs (NEB), Ipswich, MA, USA) with the respective restriction enzymes. The following New England Biolabs (MA, USA) enzymes were used: *NdeI* (R0111S), *NotI* (R3189S), *BamHI*-HF (R3136S), and *HindIII*-HF (R3104S). Digested vector was additionally dephosphorylated using Quick CIP (M0525S, NEB; MA, USA) according to the manufacturers protocol. Digested products were again purified with NucleoSpin^®^ PCR and gel clean-up kit (Macherey-Nagel, Germany). Ligation was performed using the Quick Ligation™ Kit (Cat. No. M2200, NEB, MA, USA) according to the manufacturer’s instructions. Chemically competent *Escherichia coli* (*E. coli*) DH5α cells were transformed with insert-containing plasmids by the addition of plasmid DNA into the cell suspension followed by 30 min incubation on ice. Next, cells were heat-shocked for 1 min at 42°C and again incubated for 2 min on ice. Cells were cultivated for 1 h at 37°C shaking at 200 rpm in Lysogeny broth (LB) medium (10 g/L NaCl, 5 g/L yeast extract, 10 g/L pepton/L) and plated on LB-medium agar plates, containing 30 µg/mL kanamycin. Colonies were checked for inserts in a PCR reaction using DreamTaq DNA Polymerase (Cat. No. EP0702, ThermoFisher, MA, USA). Plasmids of *E. coli* DH5α colonies positive for insertion of the respective gene were isolated with QIAprep^®^ Spin Miniprep Kit (Cat. No. 27106, Qiagen, Netherlands) and used for transformation of *E. coli* strain Rosetta pLys using the transformation protocol mentioned above, except transformed cells were cultivated on LB agar containing 30 µg/mL kanamycin and 34 µg/mL chloramphenicol. For expression of recombinant proteins, a pre-culture of *E. coli* strain Rosetta pLys cells containing *C. neoformans* genes was cultivated o.n. in LB medium containing kanamycin and chloramphenicol at 37°C, 200 rpm. 1 mL of the culture was added into fresh LB medium (with kanamycin and chloramphenicol) and grown until OD_600_ reached a value of 0.5. IPTG was added to achieve a concentration of 1mM IPTG in the culture and cells were further cultivated for 2 h at 37°C, 200 rpm. For one protein, CP_02943, a concentration of 0.5 mM IPTG was used and cells were cultivated for 4 h at 30°C for induction of protein expression. Concentration of *E. coli* cells was determined by counting before addition of IPTG and after induction of protein expression and 1x10^9^ cells were harvested, centrifuged, resuspended in Lämmli buffer (0.125 M Tris-HCl pH 6.75, 20% glycerol, 2.5% SDS, 10% 2-β-mercaptoethanol, 0.05% bromophenol blue) and boiled at 95°C for 15 min.

### Recombinant Production and Purification of Hsp71-Like Protein and Phosphoglucomutase From *C. neoformans* JEC21

The genes coding for the Hsp71-like protein and the phosphoglucomutase from *C. neoformans* serotype D strain JEC21 were amplified from cDNA using Phusion polymerase (Thermo). The primer used for amplification are listed in **Supplementary Table 1**. DNA fragments were introduced into expression plasmids by *in vitro* recombination using the InFusion HD cloning kit (Cat. No. 638920, Clontech/Takara, Saint-Germain-en-Laye, France) whereby the gene of the Hsp71-like protein was cloned into an *Nco*I restricted SM-X-URA plasmid (71) containing a sequence coding for an *N*-terminal Strep-tag and the phosphoglucomutase gene into a *BamH*I/*Not*I restricted modified pET43.1H6 plasmid introducing an *N*-terminal His-tag (72). All plasmids were initially amplified in *E. coli* DH5-α. For expression and purification of the phosphoglucomutase the pET-plasmid was transferred into *E. coli* BL21(DE3) Rosetta2 cells and expression was performed in Overnight Express Instant TB Medium (Cat. No. 71491, Novagen, Sigma-Aldrich, MO, USA). Cells were disrupted by sonication in 50 mM Tris/HCl buffer pH 8.0 with 150 mM NaCl (buffer A) and the cell-free extract was applied to a Ni-NTA Agarose gravity-flow column (2 ml bed volume, Qiagen). After a stringency wash with 20 mM imidazole in buffer A, the protein was eluted in the presence of 200 mM imidazole in buffer A and concentrated and desalted against buffer A using Amicon-Ultra centrifugal filter units with a 30 kDa cut-off (Merck, Darmstadt, Germany). The SM-X plasmid containing the Hsp71-like coding gene was used for transformation of an *Aspergillus niger* negative ATNT16 expression platform strain (71). Gene expression was induced by growth of transformants in *Aspergillus* minimal medium in the presence of 15 µg/ml doxycycline (73). Mycelium was ground to a fine powder under liquid nitrogen and resuspended in buffer A. The protein was purified to homogeneity from cell-free extracts *via* Strep-tag purification using a Strep-tactin Sepharose gravity-flow column (2 ml bed volume) as described in the manufacturer’s protocol (IBA Lifesciences). Homogeneity of purified proteins was analysed by SDS-PAGE on a NuPAGE 4-12% Bis-Tris gels in a MES-buffered running system (ThermoFisher, MA, Usa). Purified proteins were shock-frozen in liquid nitrogen and lyophilised for storage upon use.

### Quantification of Immunogenicity of Recombinant Proteins

Crude protein extracts from *E. coli* containing recombinant *C. neoformans* proteins were separated using SDS-PAGE [Owl™ Dual-Gel Vertical Electrophoresis Systems P8DS equipment (ThermoFisher Scientific, MA, USA)]. Gels contained 0.5% 2,2,2-trichloroethanol (Sigma-Aldrich, MO, USA) for staining of proteins. For protein visualization TCE-staining was UV-activated for 1 min (ChemiDoc MP, BioRad, CA, USA). Proteins were transferred from the gel onto a nitrocellulose membrane by electroblotting in tank blots using the Mini Trans-Blot equipment (BioRad, CA, USA). After blotting, membranes were blocked with 1x BlueBlock PF blocking buffer for 1.5 h (Serva, Germany) and incubated with pooled human serum (4°C, overnight), diluted 1:1,000 in 1x BlueBlock PF. Membranes were washed three times with Tris-buffered saline (TBS), containing 0.05% Tween 20 (TBS-T) and incubated (RT, 1 h) with goat anti-human IgG-AlexaFluor^®^ 647 (SouthernBiotech Cat. No. 2040-31, AL, USA) diluted 1:2,500 in BlueBlock PF, followed by three washing steps with TBS-T. Total *E. coli* proteins stained with TCE were imaged by fluorescence in the stain free channel. Immunoreactive proteins were detected subsequently in the Cy5 channel using the ChemiDoc MP device (BioRad, CA, USA). Signal intensities recorded for *E. coli* proteins (fluorescence, stain free channel) and immunoreactive signals (fluorescence, Cy5 channel) were quantified with the Image Lab 6.0.1 software (BioRad) using the “Volume tool”. The background signal, defined as signal intensity in the *E. coli* protein sample before induction of protein expression, was subtracted from the signal in the sample after induction of protein expression with IPTG. Finally, ratios of the immunoreactive signal (fluorescence, Cy5 channel) divided by the protein signal on the membrane (fluorescence, stain free channel) were calculated. Three different exposure times were analyzed per experiment. For further visualization of loading pattern and immunoreactivity quantification see **Figure 4A** and **Supplementary Figure 5**.

### Statistical Analysis

Mann–Whitney U test was used for statistical analysis of data from flow cytometry, ELISA, and quantification of immunoreactivity of recombinant cryptococcal proteins, as the data did not show a Gaussian distribution (tested by Kolmogorov–Smirnov test, D’Agostino and Pearson omnibus normality test, and Shapiro–Wilk normality test). Flow cytometry and ELISA data are presented as individual dots and medians. Data from quantification of the immunoreactivity of recombinant cryptococcal proteins is depicted as median and range. The degree of significance was annotated as following: *p ≤ 0.05, **p ≤ 0.01, ***p ≤ 0.001, ****p ≤ 0.0001. GraphPad PRISM v7 software was used for statistical analyses (GraphPad Software, La Jolla, CA, USA). Statistical analysis of the immunoproteome data was carried out as described in the 2D analysis section.

## Results

### Cryptococcal Meningitis Is Accompanied by an Increase in Anti-Cryptococcal IgG, but Not IgM Antibodies, in HIV-Negative CM Patients

We aimed to identify disease-associated proteins of *C. neoformans* that are targeted by human antibodies. Therefore, we first characterized serum samples from a Colombian cohort to (i) confirm *C. neoformans* specificity of antibodies contained in the sera, and (ii) determine the dominant antibody isotype of the human anti-cryptococcal serum antibodies. The sample collection consisted of sera from HIV-positive (CD4^+^ T cells <250 cells/µL, n=28) and HIV-negative (n=16) Colombian cryptococcal meningitis (CM) patients as well as healthy Colombian blood donors (n=15) (**Table 1**). All CM patients were diagnosed to be infected with a serotype A *C. neoformans* strain. Besides cancer or corticosteroid treatment, for most of the HIV-negative patients diagnosed with CM the underlying risk factor(s) could not be identified (termed “unknown”, **Table 1**).

**Table 1:** Collection of Colombian sera from cryptococcal meningitis patients and healthy persons.

Group	HIV status	Selection	Number of sera	Age (years)	Gender	Risk factor
Cryptococcal meningitis patients	Positive	Total sera	28	20-51; median 31	Female (n=7) Male (n=21)	HIV infection, CD4^+^ T cells <250 cells/µL.
		Quenchable sera	10	24-45 median 32	Female (n=4) Male (n=6)	HIV infection, CD4^+^ T cells <250 cells/μL.
	Negative	Total sera	16	7-72; median 42	Female (n=6) Male (n=10)	Corticosteroids: n=2; Cancer: n=3; ND: n=4; unknown: n=7.
		Quenchable sera	10	29-67 median 35	Female (n=4) Male (n=6)	Corticosteroids: n=1; Cancer: n=3; ND: n=1; unknown: n=5.
Healthy controls	Negative	Total sera	15	19-60; median 28	Female (n=8) Male (n=7)	none
		Quenchable sera	11	19-60; median 28	Female (n=6) Male (n=5)	none

Cryptococcosis patients used in this study were diagnosed with cryptococcal meningitis and were HIV-positive or HIV-negative. Control sera were derived from healthy blood donors living in Colombia. Quenchable sera were selected according to Cryptococcus neoformans specificity of the antibody signal determined by pre-absorption experiments using flow cytometry (**Supplementary Figure 1**). ND, not defined; n, Number of sera.

**Figure 1:**
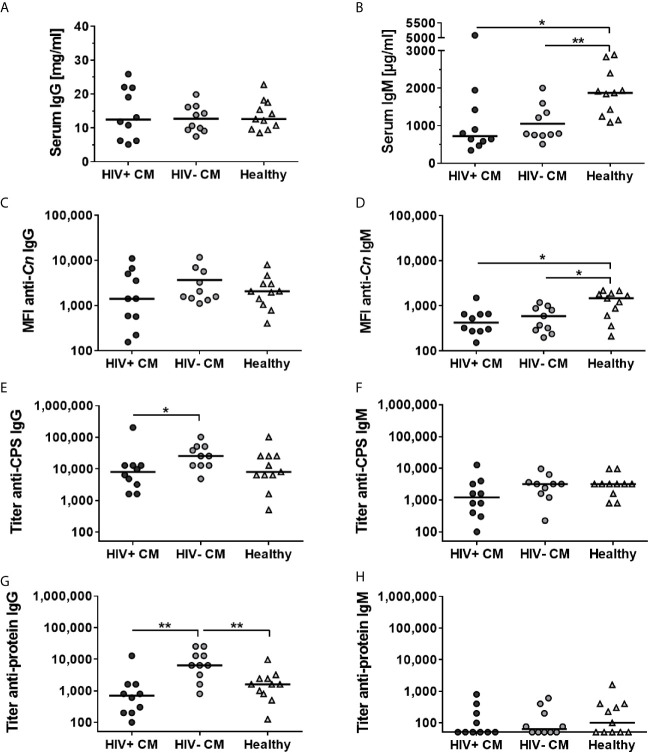
Total and *Cryptococcus neoformans*-specific IgG and IgM antibodies differ between quenchable sera from cryptococcal meningitis patients and healthy controls. Human sera from patients with cryptococcal meningitis (CM), either HIV-positive (HIV+) or HIV-negative (HIV-), and healthy control persons were analyzed. Concentration of **(A)** total serum IgG antibodies was similar in all groups, whereas concentration of **(B)** total serum IgM antibodies was increased in the healthy Colombian control group. *C. neoformans*-specific antibodies directed against intact cryptococcal cells (anti-Cn) **(C)** IgG or **(D)** IgM antibodies were quantified using flow cytometry. Median fluorescent intensity (MFI) was similar for anti-*Cn* IgG antibodies in all groups, but significantly enhanced for anti-*Cn* IgM antibodies in healthy control persons. Titers of **(E)** IgG and **(F)** IgM directed capsular polysaccharides (CPS) as well as titers of **(G)** IgG and **(H)** IgM directed against cryptococcal proteins were determined using ELISA. Titers of anti-CPS IgG and anti-protein IgG were increased in HIV-negative CM patients compared to HIV-positive CM patients and healthy control persons, whereas titers of anti-CPS IgM and anti-protein IgM were similar between all groups. Each dot represents an individual serum and lines indicate median values. Statistical analysis was carried out using the Mann–Whitney U test for comparison of two groups. Asterisks indicate significant difference.

We determined *C. neoformans* specificity of the antibodies contained in the individual sera of the collection, using a flow cytometry-based assay for quenching (74). Each individual serum was pre-absorbed with *C. neoformans* H99 (serotype A) or *Candida albicans* SC5314 cells prior to detection of IgG and IgM antibodies directed against intact cryptococcal cells (anti-*Cn* IgG and anti-*Cn* IgM). Sera that showed a significantly stronger reduction of median fluorescent intensity (MFI) for anti-*Cn* IgG and anti-*Cn* IgM when pre-absorbed with *C. neoformans* cells compared to *C. albicans* cells, were selected for further analysis (**Supplementary Figure 1** and **Table 1**). In these quenchable sera, we quantified total serum IgG and IgM concentrations (**Figures 1A, B**) and levels of anti-cryptococcal antibodies directed against (i) intact cryptococci (anti-*Cn* Igs) by flow cytometry (**Figures 1C, D**) or titers of anti-cryptococcal antibodies against (ii) purified capsular polysaccharides (CPS, anti-CPS Igs, **Figures 1E, F**), and (iii) cryptococcal proteins (anti-protein Igs, **Figures 1G, H**) by ELISA analysis. Total serum IgG levels were similar for all groups investigated (**Figure 1A**). Interestingly, levels of total serum IgM and anti-*Cn* IgM directed against intact fungal cells were significantly increased in healthy control persons, while similar among CM patients (**Figures 1B, D**). Anti-cryptococcal IgG and IgM antibodies were detected in sera of all groups, even in healthy controls at surprisingly high levels (**Figures 1C–G**). HIV-negative CM patients had higher anti-CPS IgG (**Figure 1E**) and anti-protein IgG (**Figure 1G**) titers compared to HIV-positive CM patients (CPS, proteins) and healthy control persons (proteins). This difference was also observed as a statistically non-significant trend for anti-*Cn* IgG directed against intact fungal cells (**Figure 1C**). In contrast, titers of anti-CPS (**Figure 1F**) and anti-protein IgM (**Figure 1H**) were similar for all groups, independent of HIV status or CM.

Therefore, serological data from human serum samples point towards increased production of anti-cryptococcal IgG, but not IgM antibodies, in response to cryptococcal infection. However, this was not detectable in HIV-positive CM patients, potentially due to severe immunosuppression caused by the underlying HIV infection.

### Cryptococcal Infection Results in Increased Anti-Cryptococcal IgG but Unaltered IgM Levels in a Murine Model of Pulmonary Infection

We decided to further investigate the nature of the humoral anti-cryptococcal immune response regarding (i) the dominant isotype of anti-cryptococcal antibodies, (ii) the influence of antigen dose, represented by fungal burden in the lung, and (iii) potential differences in the humoral immune response provoked by latent pulmonary infection or active systemic disease. Therefore, we used wild type (WT) Balb/c mice which are susceptible to disseminated cryptococcal infection, and IL-4Rα-deficient (IL-4Rα^-/-^) Balb/c mice which do not succumb to cryptococcal infection, but develop a latent pulmonary infection (75), after intranasal infection with the *C. neoformans* serotype D strain 1841.

**Figure 2:**
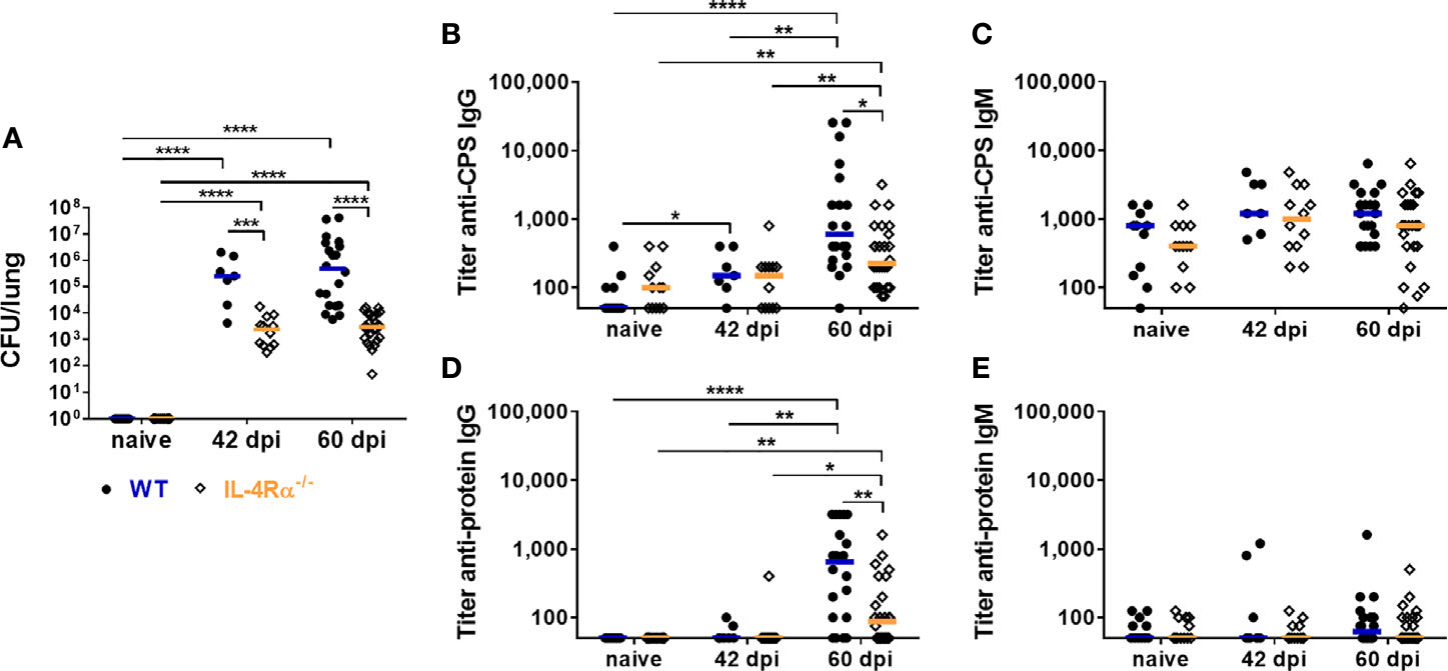
Lung fungal burden and *Cryptococcus neoformans*-specific antibody titers in wild type (WT) and IL-4Rα-deficient (IL-4Rα^-/-^) mice. WT and IL-4Rα^-/-^ mice were infected intranasally with 500 colony forming units (CFU) of *Cryptococcus neoformans* serotype D strain 1841. **(A)** Lung fungal burdens were determined in non-infected (naïve) mice, on 42 days post infection (dpi) and 60 dpi. Fungal burden were significantly higher on both 42 and 60 dpi in WT mice compared to IL-4Rα^-/-^ mice. Titers of IgG and IgM antibodies directed against capsular polysaccharides (CPS) and cryptococcal proteins were determined using ELISA analysis. Titers of **(B)** anti-CPS IgG and **(D)** anti-protein IgG increased upon infection for both genotypes, but were significantly higher in WT compared to IL−4Rα^−/−^ mice on 60 dpi. In contrast, titers of **(C)** anti-CPS IgM and **(E)** anti-protein IgM were unaffected by the progression of infection, independently of the genotype. Sera from seven to 23 mice from at least two independent experiments were analyzed per genotype. Each dot represents an individual serum and lines indicate median values. Statistical analysis was carried out using the Mann–Whitney U test for comparison of two groups. Asterisks indicate significant difference.

As previously described (75), lung fungal burdens in IL-4Rα^-/-^ mice were significantly lower on 42 days post infection (dpi) and 60 dpi, compared to WT mice (**Figure 2A**). Total serum IgG and IgM concentrations increased during the course of infection, independently of the mouse genotype (**Supplementary Figures 2A, B**). Titers of anti-cryptococcal CPS and anti-cryptoccal protein IgG and IgM antibodies were measured using ELISA analysis. Anti-cryptococcal IgG and IgM directed against the intact fungal organism (anti-*Cn* Igs) were quantified using flow cytometry. Levels of anti-cryptococcal IgG directed against each antigenic compound investigated, increased after pulmonary infection for both genotypes compared to naïve mice (**Figures 2B, D** and **Supplementary Figure 2C**). Interestingly, the increase in anti-CPS (**Figure 2B**) and anti-protein (**Figure 2D**) IgG titers was significantly higher in WT mice compared to IL-4Rα^-/-^ mice at 60 dpi (**Figures 2B, D**), but levels of anti-*Cn* IgG directed against intact cryptococcal cells were similar between both groups at all time points (**Supplementary Figure 2C**). However, titers of anti-CPS and anti-protein IgG correlated positively with lung fungal burden in WT (anti-protein IgG: r=0.5759, p=0.0017; anti-CPS IgG: r=0.4296, p=0.0253), but not in IL-4Rα^-/-^ mice (**Supplementary Table 2**). We therefore conclude that latent pulmonary infection present in IL-4Rα^-/-^ mice (75) triggers intermediate production of anti-cryptococcal IgG. In contrast, wild type mice developing disseminated cryptococcal disease (75) show further increased production of anti-cryptococcal IgG driven by increased antigen load.

In contrast to anti-cryptococcal IgG levels, anti-cryptococcal CPS and protein IgM titers were unaffected by progression of the infection (**Figures 2C, E**), with exception of elevated anti-*Cn* IgM levels in WT and IL-4Rα^-/-^ mice on day 42 post infection (**Supplementary Figure 2D**). This discrepancy might result from applying different methods for determination of anti-cryptococcal antibody levels [directed against isolated capsular material (ELISA) *vs*. intact fungal capsule (FACS)]. Surprisingly, levels of anti-*Cn* IgM antibodies directed against intact *C. neoformans* cells in WT mice were inversely correlated with lung fungal burden (r=-0.4747, p=0.0123), suggesting a decreased production of anti-*Cn* IgM antibodies with increasing fungal burden. Remarkably, anti-CPS IgM (**Figure 2C**) as well as anti-*Cn* IgM (**Supplementary Figure 2D**) antibodies were also measurable in naïve mice of both genotypes, pointing to the possibility of cross-reactivity of these antibodies with polysaccharides from other fungi, which may also be reflected in the anti-cryptococcal IgM levels measured using human serum samples (**Figures 1D, F**).

In conclusion, analysis of murine antibodies demonstrated an increase of anti-cryptococcal IgG in a lung fungal burden-dependent manner in response to cryptococcal infection, whereas this was not the case for IgM antibodies. The anti-cryptococcal IgG response was more pronounced in wild type mice, developing systemic cryptococcal disease (75), compared to IL-4Rα^-/-^ mice, that exhibit latent pulmonary infection (75).

### Immunoproteome Analysis Reveals Several Disease-Associated Cryptococcal Proteins

**Figure 3:**
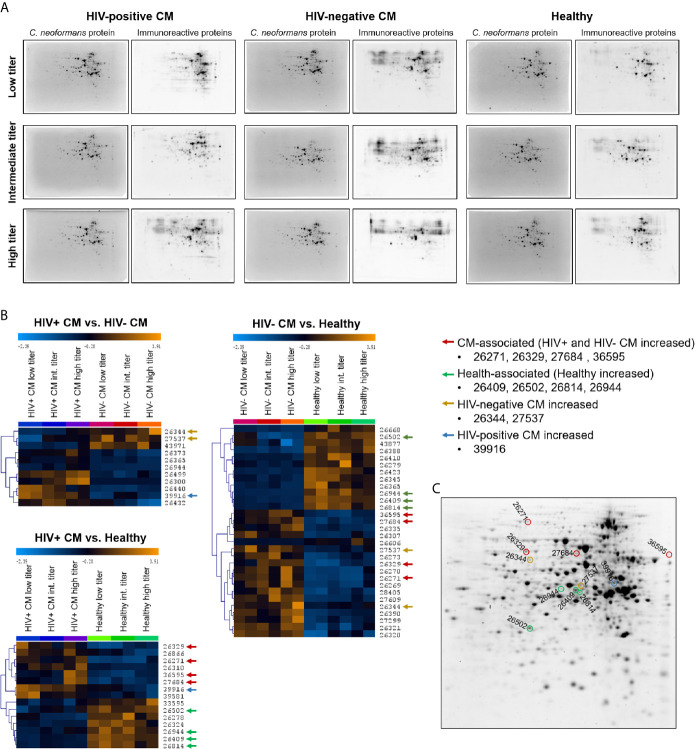
Two-dimensional (2D) analysis of the immunoproteome recognized by cryptococcal meningitis (CM) patients and healthy individuals revealed infection- and health-associated spots. *Cryptococcus neoformans* proteins (strain H99) were separated by 2D gel electrophoresis and transferred onto nitrocellulose membranes. **(A)** Representative blot images of total cryptococcal proteins (stained with UV-activated 2,2,2-trichloroethanol (TCE)) and immunoreactive protein spots bound by serum IgG (detected using AF-647 labeled secondary antibody) are shown. The contrast and brightness of the images were adjusted for publication and do not reflect actual signal intensities. **(B)** Heat maps of immunoreactive protein spots’ fluorescence signals recognized with different intensities by sera from different groups (p < 0.01). Spots were either (i) CM-associated (red arrows) – significantly stronger reactivity with CM patient sera (HIV-positive and HIV-negative) compared to healthy individuals, (ii) health-associated (green arrows) – significantly stronger reactivity with sera from healthy individuals compared to CM patients (HIV-positive and HIV-negative). Three spots were found that showed higher reactivity of (iii) HIV-negative sera (yellow arrows) or (iv) HIV-positive sera (blue arrows) compared to the respective other two groups. Immunoreactivity per sub-pool was determined in duplicates. Spot identifiers (row numbers) were automatically created by Delta 2D software. Analysis parameters: Test design: between-subjects, used Welch approximation, alpha: 0.01, p-values based on permutation, all permutations used: true, number of permutations per spot: 924, significance determined by standard Bonferroni correction, HCL: complete linkage, Euclidean Distance. **(C)** Spots of interest were highlighted in a 2D gel image. Proteins were stained with UV-activated TCE. Int., Intermediate.

Quantification of anti-cryptococcal antibodies in human and murine sera revealed IgG to be the predominant isotype induced in response to cryptococcal disease. Therefore, we decided to identify the targets of the human anti-cryptococcal protein IgG antibodies using two-dimensional (2D) gel electrophoresis and immunoblotting. Cryptococcal proteins were separated by 2D gel electrophoresis, transferred onto nitrocellulose membranes and incubated with human sera previously defined as “quenchable” sera (**Table 1**). We created serum pools for each group (HIV+ CM patients, HIV- CM patients, healthy control persons), based on the anti-protein IgG titers of the sera (low titer, intermediate titer, high titer) to facilitate detection of all proteins recognized by individual sera (**Supplementary Table 3**). Representative images of fluorescent signals of total cryptococcal proteins and immunoreactive proteins (bound by serum IgG) are shown in **Figure 3A** for all groups. Quantification with Delta 2D-software (DECODON) and subsequent statistical analysis revealed four CM-associated spots (red arrows), significantly stronger recognized by sera from both, HIV-positive and HIV-negative, CM patients, compared to healthy individuals (**Figure 3B**). Additionally, four spots were recognized with significantly higher intensity by healthy individuals (health-associated, green arrows). Two spots were strongly recognized by HIV-negative CM patients (yellow arrows) and one spot showed the highest reactivity with sera from HIV-positive CM patients (blue arrows). The respective spots are marked in a representative 2D gel image (**Figure 3C**).

Sample analysis using reverse phase chromatography coupled on-line *via* an electrospray ionization source to a mass spectrometer revealed a total of 143 proteins contained in these eleven spots of interest (**Supplementary Table 4**). Proteins were chosen for recombinant expression that were preferentially present in (i) CM-associated spots, (ii) health-associated spots, or (iii) abundant in both types of spots, indicating potential immunodominance. We expressed those proteins recombinantly to verify specific IgG-mediated recognition by human sera. cDNA sequences from *C. neoformans* strain H99 of twenty-three proteins were amplified (**Supplementary Figure 3**), cloned into pET28a+ vectors and used for transformations of *E. coli* cells. Detailed information on the proteins is listed in **Table 2**, including homology to proteins in humans and proteins of pathogenic fungi capable of causing systemic disease (*Aspergillus fumigatus*, *Histoplasma capsulatum*, *C. albicans*, *and Pneumocystis carinii*) (detailed information on homology analysis in **Supplementary Table 5**). Additionally, predicted protein function, previously reported presence in immunoreactive cryptococcal spots and evidence for extracellular localization is listed. For confirmation of successful recombinant protein expression, *E. coli* protein lysates were separated using SDS-PAGE and production of the desired proteins was confirmed by mass spectrometry of the respective protein band and staining of N- and C-terminal His-Tag on immunoblots (**Supplementary Figure 4** and **Supplementary Table 6**).

**Table 2:** Recombinant cryptococcal proteins immunoreactive with IgG from human serum samples.

Protein information Name	MW [kDa]	Accession no.	Homology					Predicted function				Described as immuno-reactive	Extra-cellular appearance
			Hu.	*Af.*	*Hc.*	*Ca.*	*Pc.*	EC description	EC no.	GO Term Name	GO ID		
26S proteasome regulatory subunit N8	38,70	AFR92184	x	x	x	x	x	Protein-serine/threonine phosphatase	3.1.3.16	No data available			
Chlorophyll synthesis pathway protein BchC	38,00	AFR97763	x	x	x	x		L-iditol 2-dehydrogenase	1.1.1.14	oxidation-reduction process	0055114		
**Cytoplasmic protein CNAG_02943**	68,78	AFR93749		x	x	x	x	No data available		No data available			Extracellular vesicle (76)
**Deoxyuridine 5’-triphosphate nucleotidohydrolase**	73,83	AFR94562	x	x	x	x	x	Histone acetyltransferase	2.3.1.48	dUMP biosynthetic process	0006226		
Extracellular elastinolytic metalloproteinase*	91,72	AFR97484		x				Metalloendo-peptidases	3.4.24.-	Metalloendo-peptidase activity	0004222		Secretory signal peptide*
**Glucose-methanol-choline oxidoreductase**	65,32	AFR94515	x	x	x			Choline dehydrogenase	1.1.99.1	oxidation-reduction process	0055114		
Glutamate dehydrogenase (NADP)	49,19	AFR97782	x	x	x	x		Glutamate dehydrogenase (NADP(+))	1.4.1.4	cellular amino acid metabolic process	0006520	(45)	Extracellular vesicle (76)
**Glycerol-3-phosphate dehydrogenase (NAD(+))**	37,80	AFR92257	x	x	x	x		Glycerol-3-phosphate dehydrogenase (NAD(+))	1.1.1.8	carbohydrate metabolic process	0005975		
**GTP-binding protein ypt1**	22,61	AFR94332	x	x	x	x	x	Small monomeric GTPase	3.6.5.2	GTPase activity	0003924		
**Heat shock 70kDa protein 4**	85,69	AFR98435	x	x	x	x	x	No data available		ATP binding	0005524		
Hsp71-like protein	69,57	AFR97929	x	x	x	x	x	Non-chaperonin molecular chaperone ATPase	3.6.4.10	ATP binding	0005524	(45, 46, 63, 64)	Extracellular vesicle (76)
Hsp72-like protein	69,51	AFR97952	x	x	x	x	x	Non-chaperonin molecular chaperone ATPase	3.6.4.10	ATP binding	0005524	(64)	
**Hsp75-like protein**	67,13	AFR92468	x	x	x	x	x	Non-chaperonin molecular chaperone ATPase	3.6.4.10	ATP binding	0005524	(45, 65)	Extracellular vesicle (76)
Hypothetical protein CNAG_05236	52,24	AFR94491		x	x	x		Fumarate hydratase	4.2.1.2	No data available			
**Hypothetical protein CNAG_06113**	36,61	AFR98337		x	x	x		No data available		RNA binding	0003723		Extracellular vesicle (76)
Hypothetical protein CNAG_06946	39,13	AFR94883	x	x	x	x	x	No data available		No data available			
Ketol-acid reductoisomerase, mitochondrial	44,34	AFR96043		x	x	x		Ketol-acid reductoisomerase (NADP(+))	1.1.1.86	branched-chain amino acid biosynthetic process	0009082	(65, 66)	Extracellular vesicle (76)
Mannose-1-phosphate guanyltransferase	39,95	AFR98009	x	x	x	x	x	Mannose-1-phosphate guanylyltransferase	2.7.7.13	GDP-mannose biosynthetic process	0009298	(65)	
**Phosphoglucomutase**	60,54	AFR98550	x	x	x	x	x	Phosphoglucosamine mutase	5.4.2.10	carbohydrate metabolic process	0005975	(64)	
Pyruvate decarboxylase	67,61	AFR97558		x	x	x		Pyruvate decarboxylase	4.1.1.1	mitochondrion	0005739	(64)	Extracellular vesicle (76)
**Transaldolase**	35,29	AFR98178	x	x	x	x		Transaldolase	2.2.1.2	carbohydrate metabolic process	0005975	(46, 63, 64)	
Transketolase	74,33	AFR95182	x	x	x	x	x	Transketolase	2.2.1.1	transketolase activity	0004802		
**Urease accessory protein UreG**	33,63	AFR92807		x	x			No data available		nitrogen compound metabolic process	0006807		

Twenty-three cryptococcal proteins were recombinantly expressed in Escherichia coli. Production of the desired protein was confirmed using mass spectrometry. All proteins were proven to be immunoreactive with human sera. Proteins printed in bold were classified as disease-associated cryptococcal proteins, as they showed significantly stronger reactivity with sera from cryptococcal meningitis patients compared to healthy individuals on Western Blots. Presence of a secretory signal peptide was checked with SignalP-5.0*. Detailed information on homology to human proteins (Hu.), or homologous proteins in fungal pathogens causing systemic infections such as Aspergillus fumigatus (Af.), Histoplasma capsulatum (Hc.), Candida albicans (Ca.), and Pneumocystis carinii (Pc.) are listed in **Supplementary Table 4**. Predicted function was collected from the database FungiDB^#^ which uses orthology to predict gene function. Enzyme Commission (EC) numbers (no.), classifying enzymes based on the chemical reactions they catalyze and corresponding descriptions are listed. Additionally, Gene Ontology (GO) Term Names, describing predicted biological processes, cellular localization, or molecular functions the protein may be involved with and corresponding GO identifier (GO ID) are included. Information on previous description of the respective protein to be contained in immunoreactive spots as well as evidence for extracellular appearance is listed in the table.

*http://www.cbs.dtu.dk/services/SignalP/.

^#^ https://fungidb.org/fungidb/app.

**Figure 4:**
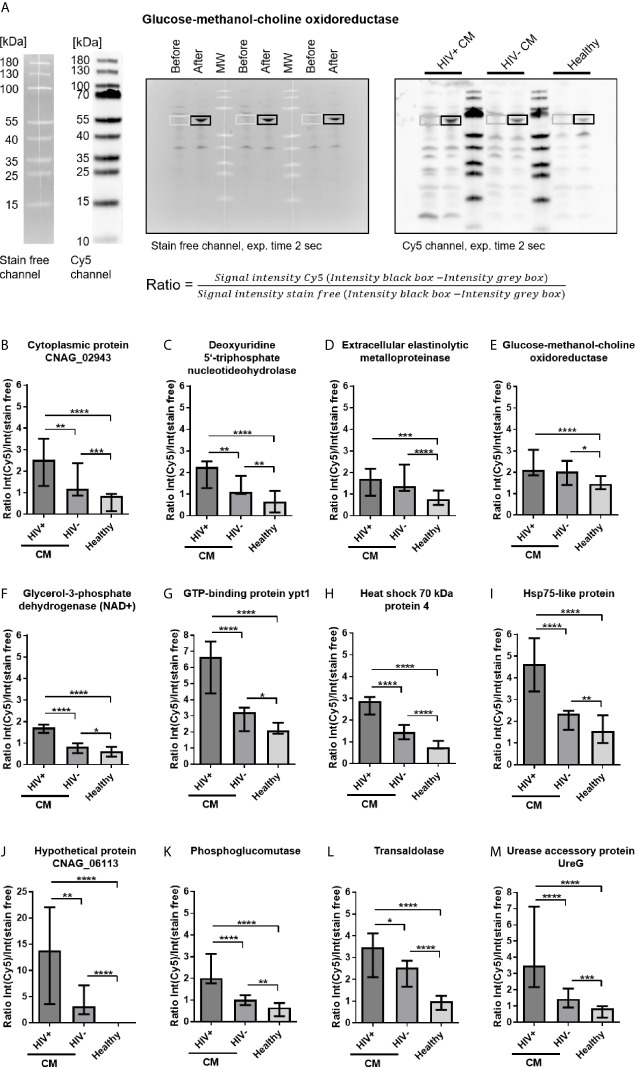
Cryptococcal meningits (CM)-associated recombinant cryptococcal proteins reactive with serum IgG. **(A)** *Escherichia coli* lysates containing recombinant cryptococcal proteins were separated by SDS-PAGE. Samples before induction of recombinant protein expression and samples after induction of protein expression using IPTG were loaded, separated by MW markers, as representatively shown for glucose-methanol-choline oxidoreductase. *E. coli* proteins were blotted onto nitrocellulose membranes and (left) stained with 2,2,2-trichloroethanol (TCE, detection in stain free channel). Membranes were incubated with pooled sera from HIV-positive (HIV+) CM patients, HIV-negative (HIV-) CM patients, or healthy control persons, as marked in the blot. (Right) Proteins immunoreactive with human IgG were detected using polyclonal goat-anti human IgG coupled to Alexa Fluor 647 (AF-647, detected in Cy5 channel). Ratios of fluorescence intensities of the respective area in both channels were calculated to normalize the immunosignal (Cy5 channel) onto the amount of protein loaded (stain free channel). **(B–M)** CM-associated cryptococcal proteins reactive with serum IgG are shown. Proteins depicted showed significantly stronger reactivity with sera from CM patients (HIV+ and HIV-) compared to sera from healthy individuals. Median values and range of three independent experiments, comprising values from three different exposure times for each experiment, are shown. Statistical analysis was carried out using the Mann–Whitney U test for comparison of two groups. Asterisks indicate significant difference. MW, Molecular weight marker; exp., exposure.

For quantification of recombinant protein immunoreactivity, *E. coli* proteins were separated by SDS-PAGE, blotted onto nitrocellulose membranes and incubated with pools of sera from HIV-positive CM patients, HIV-negative CM patients, or healthy control persons. Based on similar 2D immunoproteome analysis results (**Figure 3B**) obtained for all three serum sub-pools of each group (low, intermediate, and high titer, **Supplementary Table 2**), all sera of each respective group (HIV+CM, HIV-CM, Healthy) were pooled for quantifying the immunoreactivity of the recombinant proteins. Quantification of immunoreactivity was performed as shown in **Figure 4A**: *E. coli* samples before induction of recombinant protein expression and after induction of recombinant protein expression using IPTG were loaded side by side. For serum incubation, the blots were split in three parts and incubated with the indicated serum pools. For calculation, background signal intensity before induction of protein expression (grey boxes) was subtracted from signal intensity after induction of protein expression (black boxes). Finally, the immunoreactive signal intensity (fluorescence, Cy5 channel) was divided by the signal intensity of *E. coli* protein (fluorescence, stain free channel), thereby normalizing the immunoreactivity onto the protein loading. Representative blots for all proteins are shown in **Supplementary Figure 5**. Using this approach, twenty-three proteins were confirmed to be immunoreactive with human sera (**Figure 4** and **Supplementary Figure 6**). Most proteins showed reactivity with sera from HIV-positive and HIV-negative CM patients, but also with sera from healthy individuals.

Our screen revealed twelve disease-associated cryptococcal proteins, as the recombinant proteins showed significantly stronger reactivity with serum IgG from CM patients compared to healthy control persons (**Figures 4B–M**). Two proteins, extracellular elastinolytic metalloprotease and glucose-methanol-choline oxidoreductase, showed similar reactivity in both HIV-positive and HIV-negative CM patients (**Figures 4D, E**). The remaining ten proteins were recognized significantly stronger by serum IgG from HIV-positive CM patients compared to HIV-negative CM patients (**Figures 4B, C**, **F–M**). Eleven recombinant cryptococcal proteins were proven to be immunoreactive with human sera, although with different recognition patterns: Five proteins were preferentially immunoreactive with sera from HIV-positive CM patients (**Supplementary Figures 6A–E**). Four proteins showed decreased reactivity with sera from HIV-negative CM patients (**Supplementary Figures 6F–I**), and two proteins were recognized with similar intensities by all three groups (**Supplementary Figures 6J, K**). Among all recombinant proteins, four proteins showed remarkably strong immunoreactivity with sera of all groups (Ratio value >2 for all groups). These proteins, GTP-binding protein ypt1 (**Figure 4G**), Hsp71-like protein (**Supplementary Figure 6B**), Hsp72-like protein (**Supplementary Figure 6J**) and ketol-acid reductoisomerase (**Supplementary Figure 6I**), could therefore represent immunodominant proteins. Two additionally produced recombinant proteins derived from the gene sequence of serotype D strain *C. neoformans* JEC21, Hsp71-like protein purified from a heterologous expression in *Aspergillus niger* and purified phosphoglucomutase expressed in *E. coli*, were also proven to be immunoreactive with sera from CM patients and healthy individuals (data not shown), although we did not perform quantification of immunoreactivity for these proteins. Both proteins show very high homology to their corresponding homologue in the serotype A strain H99 (**Supplementary Table 7**). This indicates cross-reactivity between corresponding proteins from different *C. neoformans* serotypes.

## Discussion

In this study, we characterized the humoral immune response in HIV-positive and HIV-negative CM patients as well as healthy individuals regarding (i) the quantity of anti-cryptococcal IgG and IgM antibodies to identify the dominant isotype in anti-cryptococcal humoral immunity, and (ii) the target proteins of the human humoral immune response against *C. neoformans* to identify disease-associated cryptococcal proteins.

Our study revealed IgG to be the dominant isotype induced in response to CM. This was reflected by increased titers of anti-cryptococcal protein and CPS IgG in HIV-negative CM patients compared to HIV-positive CM patients and healthy individuals, although not reaching statistical significance for anti-CPS IgG (HIV-negative CM patients compared to healthy group). Similarly, previous studies showed increased anti-glucuronoxylomannan (GXM) titers in HIV-negative CM patients compared to healthy individuals (19) or HIV-positive cryptococcosis patients (61). However, in our study similar titers of anti-cryptococcal IgG antibody were detected in HIV-positive CM patients and healthy individuals, confirming previous reports (55, 58, 62). We hypothesize, that severely immunosuppressed AIDS patients (CD4^+^ T cell count <250 cells/µL) are not able to mount a proper immune response to cryptococcal infection, and, therefore, no increase in anti-cryptococcal IgG titers is detectable in these patients. However, some studies measured increased anti-GXM IgG titers in HIV-positive patients with or without cryptococcal infection compared to HIV-negative individuals (49, 50, 52, 53, 56). This might be caused by underlying HIV infection leading to increased levels of serum IgG in general (77–79). In the sera used here, however, total serum IgG concentrations were similar in all groups (**Figure 1A**). Corroborating the data from HIV-negative CM patients, cryptococcal infection of WT and IL-4Rα^-/-^ mice led to an increase in anti-cryptococcal IgG, but not IgM production, consistent with a previously published study (80). Interestingly, titers of anti-cryptococcal IgG were significantly higher in WT mice, developing disseminated cryptococcal disease and high fungal burden in the lung (75), compared to IL-4Rα^-/-^ mice, exhibiting a latent pulmonary cryptococcal infection without overt disease (75).

Remarkably, anti-cryptococcal IgM and IgG antibodies were detected in considerably high frequencies even in sera from healthy human individuals, which we categorize as “pre-existing” antibodies. This finding is in accordance with previously published results, demonstrating the presence of antibodies directed against cryptococcal CPS (33, 47–54, 56), cryptococcal proteins (58–62) or mannoproteins (57) in human sera, independent of cryptococcal disease or the HIV status.

We therefore hypothesize that latent pulmonary infection, as observed in IL-4Rα^-/-^ mice (75) and hypothesized in healthy individuals evidenced by reactivation of dormant cryptococcal infection (81, 82), is sufficient to trigger basal production of anti-cryptococcal IgG and IgM antibodies. Alternatively, environmental exposure of humans to cryptococcal cells detectable in different reservoirs (3–6) could trigger the observed production of anti-cryptococcal Igs. In contrast, dissemination of *C. neoformans* leading to systemic cryptococcal disease, as present in WT mice (75) and CM patients, leads to an increase in anti-cryptococcal IgG, but not IgM antibodies. However, in CM patients suffering from severe immunosuppression (CD4^+^ T cells <250 cells/µL), production of anti-cryptococcal IgG antibodies in response to infection may be impaired. Anti-cryptococcal IgM antibodies ubiquitously present in the sera analyzed in this study are believed to mainly target polysaccharides of the cell wall, which are conserved structures (83) ubiquitously present on fungal organisms colonizing mice and humans (84). Therefore, those antibodies may be cross-reactive with different fungal species and could be non-specific for *C. neoformans*, as evidenced by the presence of anti-CPS IgM titers in naïve mice in our study and additional studies (33, 80, 85), and thus could potentially be produced in response to colonization by commensal fungi.

Interestingly, total serum IgM levels were found to be decreased in HIV-positive and HIV-negative CM patients compared to healthy individuals, which has also been observed by McGowan et al. in 2006 (77), although other studies demonstrated increased serum IgM in HIV-positive patients compared to healthy individuals (52, 53, 78). Anti-*Cn* IgM antibodies directed against intact cryptococcal cells were also present at significantly lower levels in CM patients compared to healthy control persons. Interestingly, previous studies showed lower percentage of IgM-expressing memory B cells compared to healthy individuals in both HIV-positive (55) and HIV-negative (19) cryptococcosis patients, proposing a decreased proportion of IgM-expressing memory B cells as a risk factor for cryptococcal disease, indicating a role of IgM antibodies in defense against *C. neoformans*.

We aimed to identify cryptococcal proteins targeted by human serum IgG antibodies, as previous studies demonstrated protection of mice from lethal challenge with *C. neoformans* after immunization with protein fractions immunoreactive with mouse sera (45, 46), or proteins with immunogenic properties packaged into glucan particles (86, 87). Additionally, we aimed to identify disease-specific proteins, exclusively recognized by sera from CM patients, indicating a role in cryptococcal pathogenesis and therefore rendering them potential targets of anti-cryptococcal therapy.

Immunoreactivity of twenty-three cryptococcal proteins contained in immunoreactive protein spots was verified by recombinant expression and subsequent incubation with human sera. Most proteins showed reactivity with sera of all groups (HIV-positive and HIV-negative CM patients, as well as healthy individuals). Surprisingly, immunoreactivity of most recombinant proteins was highest with sera from HIV-positive CM patients, in contrast to anti-cryptococcal IgG titers in those patients, which were similar to titers in healthy individuals and higher in HIV-negative CM patients. Recognition of major immunogenic antigens by anti-cryptococcal antibodies may be facilitated when specific proteins are used in contrast to crude antigenic mixtures utilized for ELISA analysis, containing limited amounts of specific antigens.

Twelve proteins were demonstrated to be disease-associated, as they were significantly stronger recognized by sera from CM patients compared to healthy persons. Four of those proteins are especially interesting candidates for vaccine development, as they do not show homology to any known human protein, but to proteins from other pathogenic fungi. Those four proteins are extracellular elastinolytic metalloprotease, cytoplasmic protein CNAG_02943, hypothetical protein HP_06113, and urease accessory protein UreG.

Extracellular elastinolytic metalloproteinase is critical for crossing of the blood-brain barrier and therefore required for establishment of fungal disease in the central nervous system. This was demonstrated using an *mpr1Δ C. neoformans* strain, with *mpr1* being the serotype D homologue of the elastinolytic metalloprotease identified in our study (88, 89). Extracellular elastinolytic metalloprotease is an especially interesting target protein for development of anti-cryptococcal treatment options, as inhibition of Mpr1 by natural product inhibitors prevented cryptococcal cells from crossing the blood brain barrier in an *in vitro* transwell model (90). Extracellular elastinolytic metalloproteinase contains a secretory signal peptide making it also an attractive candidate for vaccine development. Indeed, vaccination with recombinant extracellular elastinolytic metalloprotease (Mep1) contained in glucan particles led to significantly prolonged survival of C57BL/6 mice when challenged orotracheally into the lung with *C. neoformans* H99 (87).

Two disease-associated proteins with unknown functions, cytoplasmic protein CNAG_02943 and hypothetical protein CNAG_06113, predicted to possess RNA-binding capacity, have been identified in our screen. Interestingly, both proteins were detected in extracellular vesicles of *C. neoformans*, implicating them in cryptococcal virulence (76) and also rendering them interesting vaccine candidates.

Urease accessory protein UreG is required for activation of apourease (91), an enzyme crucial for cryptococcal virulence, as urease hydrolyzes urea for usage as nitrogen source and leading to increased local pH interfering with host function (92). UreG mediates activation of apourease by incorporation of Ni^2+^ ions, and was shown to be critical for brain invasion, as a mutant strain lacking *ure7*, encoding UreG in *C. neoformans*, led to significantly reduced fungal burden in brains of C57BL/6 mice infected intravenously with *ure7Δ* or wild type H99 cryptococcal cells (91). Therefore, urease accessory protein UreG is a promising target for anti-cryptococcal chemotherapeutics.

We identified eight additional disease-associated cryptococcal proteins. All those proteins possess homologs in *Homo sapiens*, although with varying sequence similarity (for further information see **Supplementary Table 4**). Some of these proteins may nonetheless represent interesting targets for development of anti-cryptococcal therapy, based on their roles in cryptococcal metabolism, virulence or survival.

Phosphoglucomutase, transaldolase, and glycerol-3-phosphate dehydrogenase (NAD+) are confirmed or predicted to be central metabolic enzymes involved in carbohydrate metabolism (93). Two of them were previously described to be contained in *C. neoformans* protein spots immunoreactive with sera from mice infected with *C. neoformans* strain 1841 (phosphoglucomutase, transaldolase) (64), or sera from mice immunized with the *C. neoformans* strain H99γ, engineered to produce murine IFN-γ, or immunized with *C. gattii* protein fractions (transaldolase) (46, 63). Additionally, phosphoglucomutase derived from the genetic sequence of cryptococcal serotype D strain JEC21 was also recognized by human sera, indicating serotype-independent recognition. Interestingly, transaldolase is implicated in virulence of *C. neoformans* as it possesses the capability to bind heparin (94) and plasminogen (95) and shows increased expression in response to nitric oxide stress (96). Another disease-associated protein identified in our screen, the enzyme glucose-methanol-choline oxidoreductase, was shown to be upregulated in *C. gattii* under iron deprivation, implicating a role in iron acquisition which is critical for cryptococcal pathogenesis (97). The homologues of the disease-associated protein GTP-binding protein ypt1 in *C. albicans* (98), and *Saccharomyces cerevisiae* (99, 100) are critical for intracellular vesicle traffic and cell survival, as null mutants of *S. cerevisiae* are not viable (101).

Four *C. neoformans* heat shock proteins of the Hsp70 family were proven to be immunoreactive in our study. This confirms previous studies, revealing Hsp70 proteins to be contained in cryptococcal protein spots reactive with sera from patients with pulmonary cryptococcosis (102), mice immunized with the *C. neoformans* strain H99γ (45, 63), mice intratracheally infected with *C. neoformans* strain YC-11 (serotype A) (103), or 1841 (serotype D) (64), mice immunized with *C. gattii* protein fractions (46), and koalas infected with *C. gattii* (65). Two Hsp70 proteins identified to be immunoreactive in our study, Hsp71-like protein and Hsp75-like protein, were also detected in extracellular vesicles of *C. neoformans* (76). Furthermore, anti-Hsp70 antibodies, directed against cryptococcal Hsp70 protein recombinantly produced in *E. coli*, revealed that Hsp70 proteins are located at the fungal surface (104). This indicates extracellular presence of Hsp70 proteins rendering them interesting antigens for vaccine development. Furthermore, the heat shock protein Ssa1, corresponding to Hsp71-like protein in our study, is implicated in fungal virulence by influencing the immune response towards M2 macrophage polarization during early infection in a pulmonary mouse infection model (105). In our study, Hsp75-like protein and Hsp70 protein 4 were demonstrated to be disease-associated, whereas Hsp71-like and Hsp72-like proteins were preferentially recognized by HIV-positive, but not HIV-negative patients compared to healthy individuals. Additionally, we could demonstrate serotype-independent recognition of Hsp71-like protein by human sera. Overall, proteins of the Hsp70 family showed particularly strong immunoreactivity with all sera, emphasizing their immunodominant role previously described.

In conclusion, we identify several disease-associated cryptococcal protein antigens based on their preferential reactivity with human sera from HIV-positive and HIV-negative CM patients. Some of these proteins are interesting candidates for future research on anti-cryptococcal chemotherapy or development of an anti-cryptococcal vaccine. Several proteins are implicated in cryptococcal virulence or fungal metabolism and survival and could therefore be targeted by anti-fungal agents. One disease-associated protein identified in our screen, extracellular elastinolytic protease, was already successfully inhibited by natural products in an *in vitro* transwell model, decreasing cryptococcal virulence (90). Therefore, targeting other virulence-associated proteins using similar approaches could be beneficial. Potential candidate antigens for development of anti-cryptococcal vaccines should be located on the cell surface or presented extracellularly to facilitate antibody-mediated neutralization and recognition by antigen-presenting cells. Extracellular location is evident for seven immunoreactive proteins identified in our screen, rendering them promising candidates for a vaccination approach. Furthermore, some of these proteins did not show homology to human proteins, and are therefore excellent targets for further development of an anti-cryptococcal vaccine, as this minimizes the risk of autoimmune responses (26). Additionally, all disease-associated proteins identified in our screen possess homologous proteins in other fungal pathogens, rendering them potential targets for development of pan-fungal vaccines (26).

## Data Availability Statement

The original contributions presented in the study are included in the article/**Supplementary Material**. Further inquiries can be directed to the corresponding authors.

## Ethics Statement

The studies involving human participants were reviewed and approved by Technical Committee of Research (CTIN) and the Ethical Committee for Research (CEIN) of the National Institute of Health, Bogota, Colombia; Ethical committee of Corporación para Investigaciones Biológicas (CIB) and Hospital La Maria IRB Number 7250 in Medellin, Colombia. Written informed consent to participate in this study was provided by the participants’ legal guardian/next of kin.

## Author Contributions

AEG designed experiments, performed experiments and wrote the manuscript. DV, UM, and MB designed and performed experiments. CF designed the experiments and provided serum samples. CS and BS-R developed methods. FB, PE, and RH provided mice, serum samples and key reagents. GA conceptualized the project and wrote the manuscript. All authors contributed to the article and approved the submitted version.

## Funding

AEG was supported by funds from the Ph.D. program (“Doktorandenförderplatz”) of the University of Leipzig, Leipzig, Germany. CF was supported by a Georg Forster Research Fellowship for postdoctoral researchers from the Alexander von Humboldt Foundation. Equipment and software for mass spectrometry analysis was funded by the European Fund for Regional Structure Development (grant number 100193542) and the Deutsche Forschungsgemeinschaft (grant number INST 268/387-1). We acknowledge support from Leipzig University for Open Access Publishing.

## Conflict of Interest

The authors declare that the research was conducted in the absence of any commercial or financial relationships that could be construed as a potential conflict of interest.

## Publisher’s Note

All claims expressed in this article are solely those of the authors and do not necessarily represent those of their affiliated organizations, or those of the publisher, the editors and the reviewers. Any product that may be evaluated in this article, or claim that may be made by its manufacturer, is not guaranteed or endorsed by the publisher.
