# Supplementary figures and images for: Identification of Disease-Associated Cryptococcal Proteins Reactive With Serum IgG From Cryptococcal Meningitis Patients
Source: Front Immunol. 2021 Jul 23;12:709695. doi: 10.3389/fimmu.2021.709695 (PMC8342929; doi:10.3389/fimmu.2021.709695)

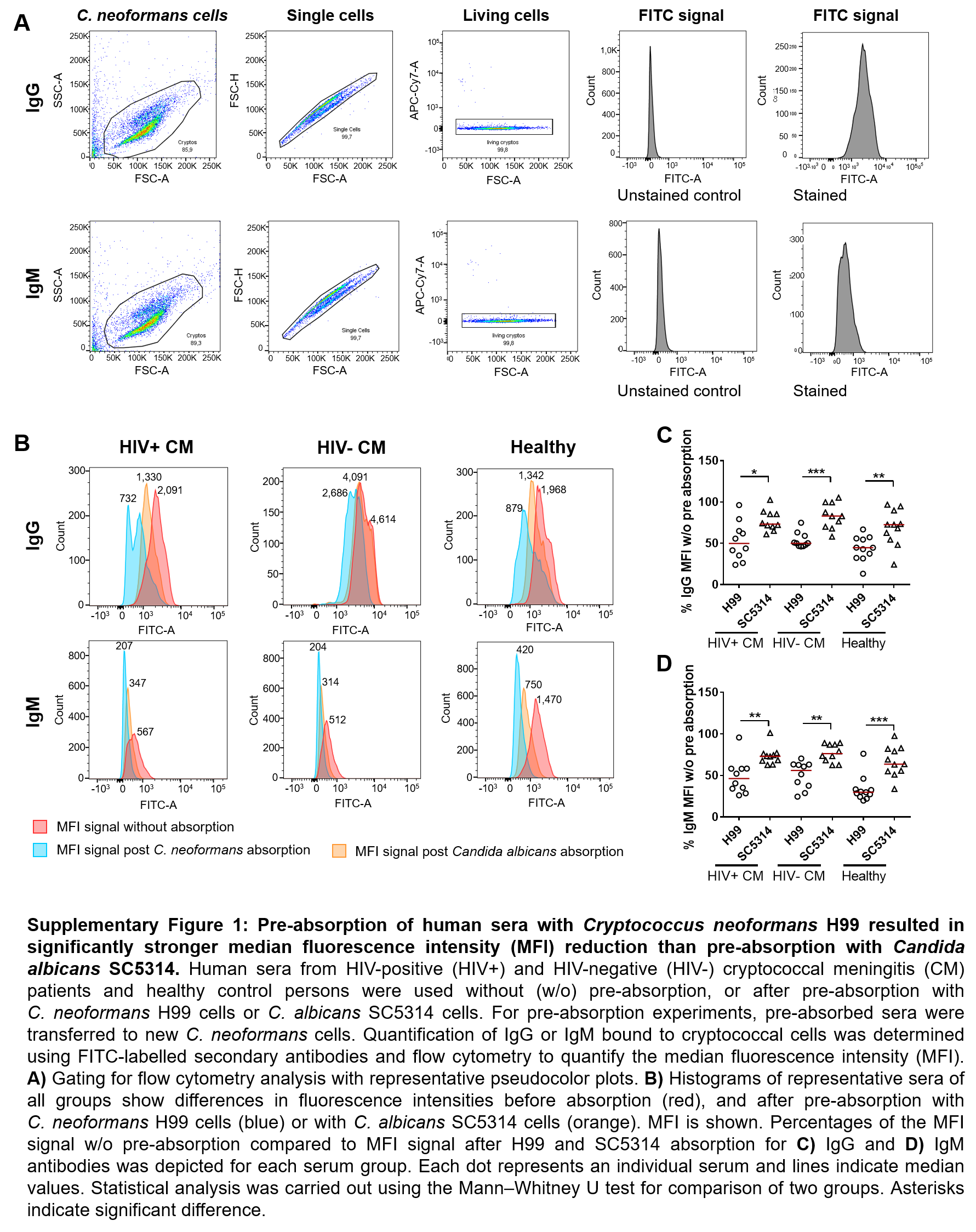

Supplement: Supplementary file 1 [file Image_1.tif]

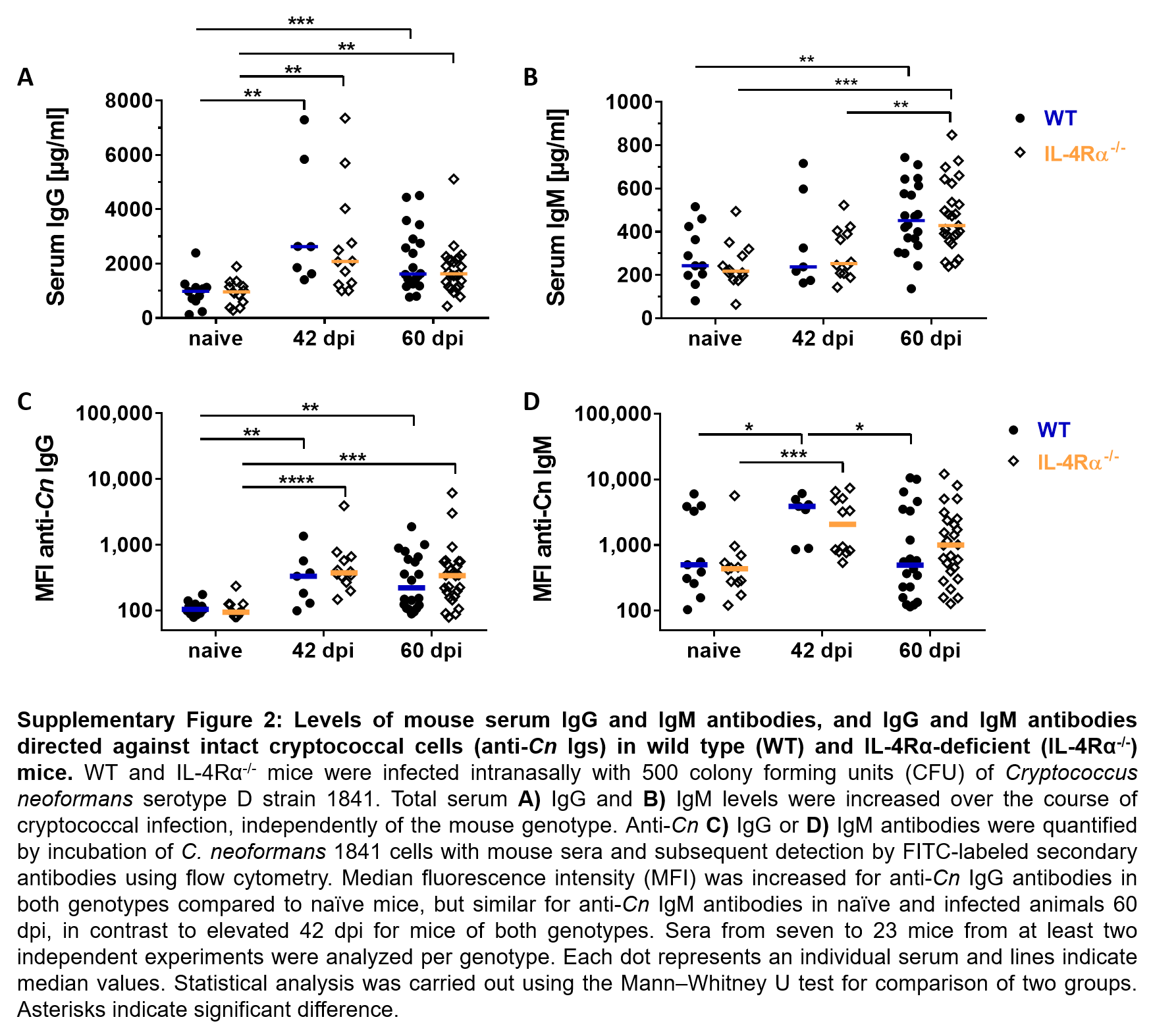

Supplement: Supplementary file 2 [file Image_2.tif]

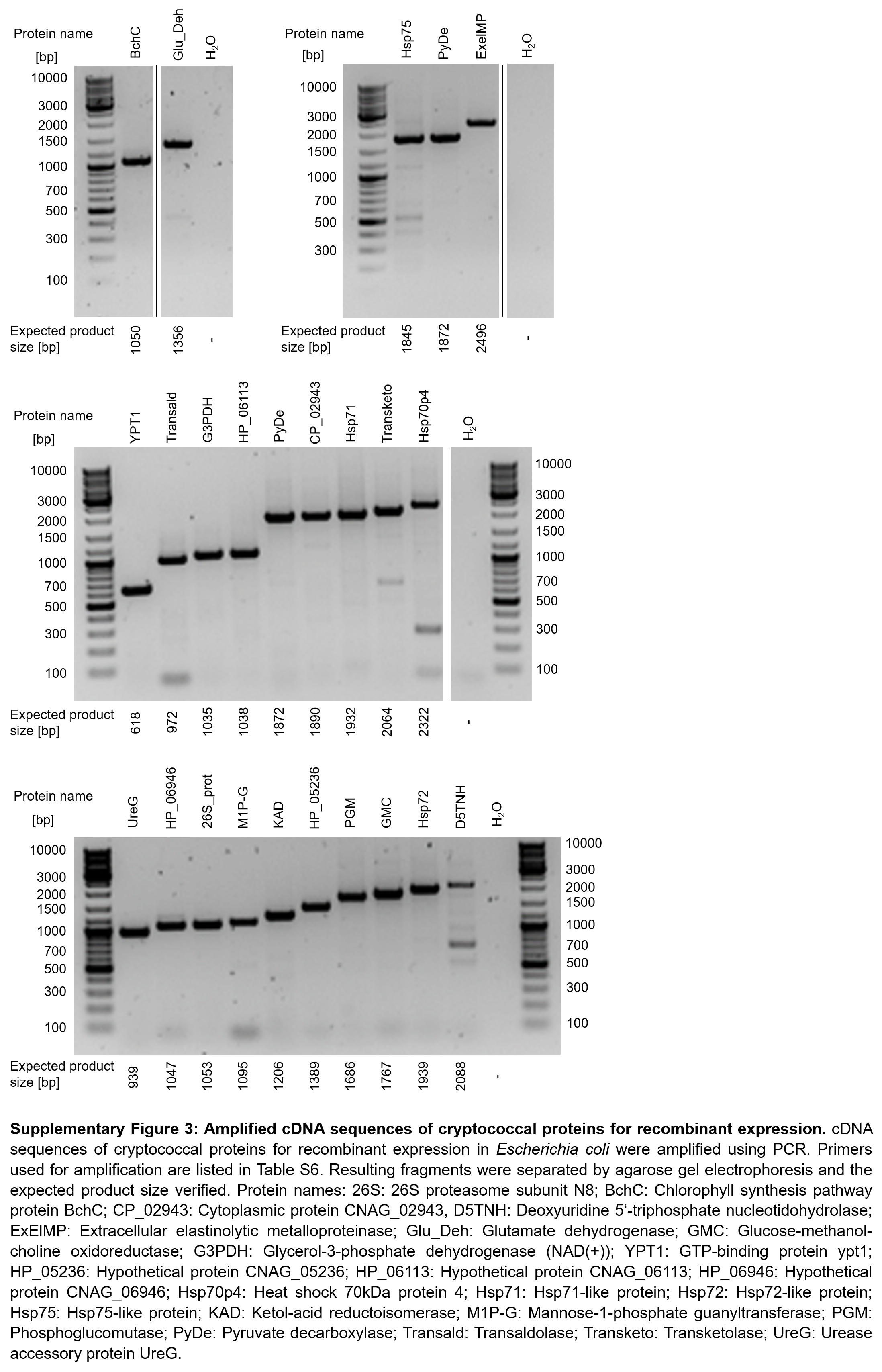

Supplement: Supplementary file 3 [file Image_3.tif]

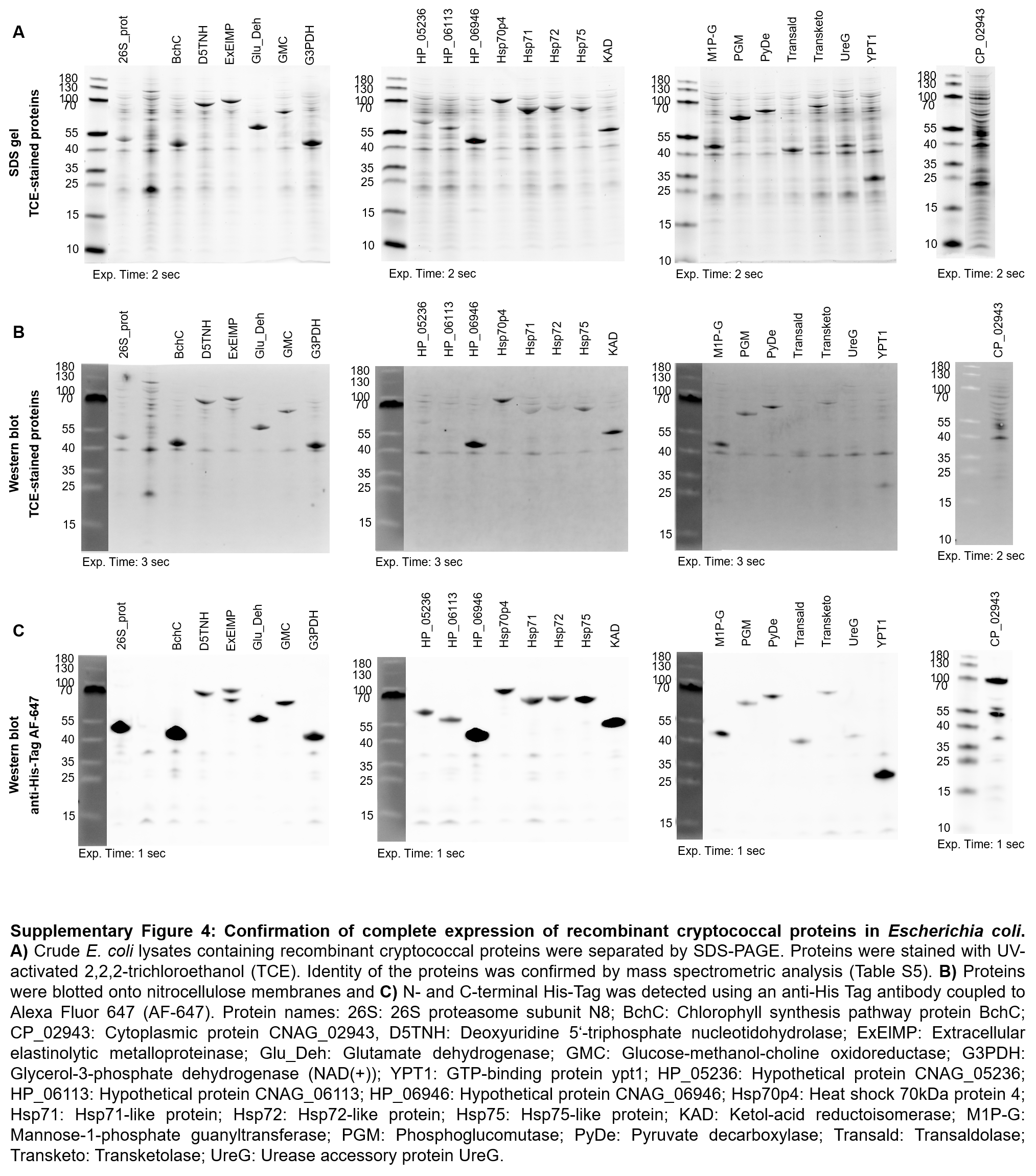

Supplement: Supplementary file 4 [file Image_4.tif]

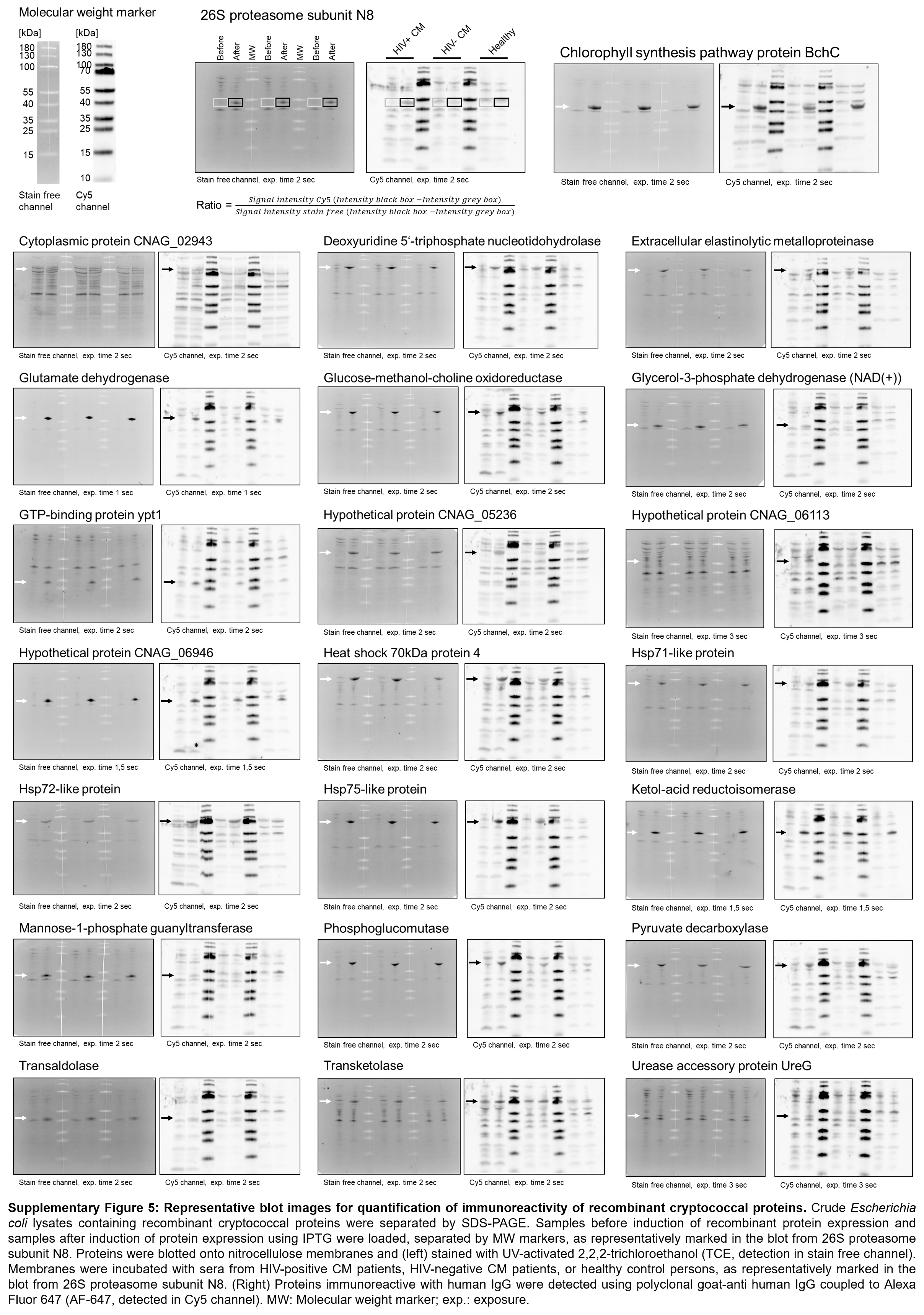

Supplement: Supplementary file 5 [file Image_5.tif]

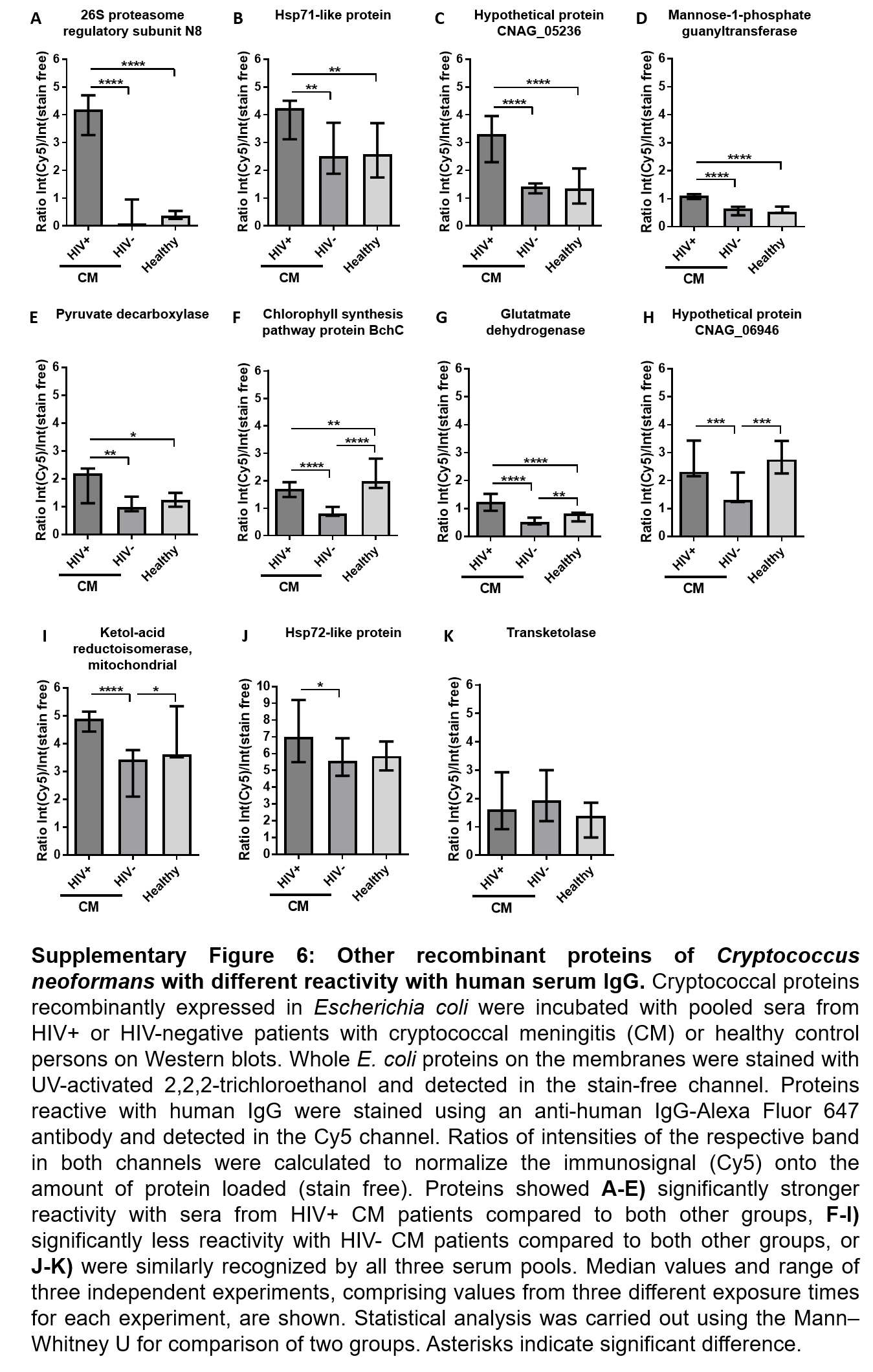

Supplement: Supplementary file 6 [file Image_6.tif]
