# Supplementary material for: Identification of Disease-Associated Cryptococcal Proteins Reactive With Serum IgG From Cryptococcal Meningitis Patients
Source: Front Immunol. 2021 Jul 23;12:709695. doi: 10.3389/fimmu.2021.709695 (PMC8342929; doi:10.3389/fimmu.2021.709695)
Supplement: Supplementary file 7 [file Table_1.docx]

Supplementary Material

# Supplementary Table 1

**Supplementary table 1: Primer sequences** used for amplification of cDNA sequences from *C. neoformans* for recombinant protein expression.

| **Targeted Protein** | **Serotype / strain** | **Primer name** | **Sequence** |
| --- | --- | --- | --- |
| 26S proteasome regulatory subunit N8 | serotype A / H99 | 26S_prot_fwd | TT ACT CAT ATG CCC GGC TTA ACA ACG GCA C |
| 26S proteasome regulatory subunit N8 | serotype A / H99 | 26S_prot_rev | CGT TTT GCGG CCGC CTT TTT CTT CTT CTC TTT CTC CTC T |
| chlorophyll synthesis pathway protein BchC | serotype A / H99 | Chloro_BchC_fwd | GC ACC CAT ATG GTC GCC AAG GAG ATG AAC G |
| chlorophyll synthesis pathway protein BchC | serotype A / H99 | Chloro_BchC_rev | GCT TTT GCG GCC GCG TCC TTG TGT TCG GGC TTG |
| Cytoplasmic protein CNAG_02943 | serotype A / H99 | CP_02943_rev | GGC CCT GCGG CCGC CTC CTT TTT GGC TGA TCC AAA GT |
| Cytoplasmic protein CNAG_02943 | serotype A / H99 | CP_02943_fwd | TAT TC CAT ATG TCC CAT TTC GAC ACT GTC TCC |
| deoxyuridine 5~-triphosphate nucleotidohydrolase | serotype A / H99 | D5TNH_fwd | TA TTA CAT ATG TCC AGA TTC GTC AGG CCT TC |
| deoxyuridine 5~-triphosphate nucleotidohydrolase | serotype A / H99 | D5TNH_rev | GTA AAA GCGG CCGC AAT CAA GCT CCC AGC AAC ATC |
| extracellular elastinolytic metalloproteinase | serotype A / H99 | Ex_el_MP_fwd | TA TAA CAT ATG CGC TCC TCC GCG CTC AT |
| extracellular elastinolytic metalloproteinase | serotype A / H99 | Ex_el_MP_rev | CGA TAA GCG GCC GCA GCC TTT TTG GAC TCG CAG AC |
| glucose-methanol-choline oxidoreductase | serotype A / H99 | GMC_oxired_fwd | TA TTA CAT ATG GTT CAC GCT GCT ACT CAC C |
| glucose-methanol-choline oxidoreductase | serotype A / H99 | GMC_oxired_rev | AA CCC AAG CTT CTT TGT CTC TTT GTA AAG GTC GG |
| glutamate dehydrogenase (NADP) | serotype A / H99 | Glu_Dehyd_rev | GCA TTT GCG GCC GCC CAC CAG TCA CCC TGT TCG |
| glutamate dehydrogenase (NADP) | serotype A / H99 | Glu_Dehyd_fwd | TA TTA CAT ATG TCC AAC TAC CCC TCT GAG CC |
| glycerol-3-phosphate dehydrogenase (NAD(+)) | serotype A / H99 | GPDH_2_fwd | TA TGG CAT ATG GGC AAG GAA AAG GTT GCT GTT |
| glycerol-3-phosphate dehydrogenase (NAD(+)) | serotype A / H99 | GPDH_2_rev | GCA AAT GCGG CCGC AAG CCC CTC GGT CAG TTT C |
| GTP-binding protein ypt1 | serotype A / H99 | YPT1_rev | ACT AAA GCGG CCGC GCA GCA TCC ACC AGC GGT |
| GTP-binding protein ypt1 | serotype A / H99 | YPT1_fwd | TA TAA CAT ATG TCT GCC CCA GAA TAC GAC TAC |
| heat shock 70kDa protein 4 | serotype A / H99 | HSP70_P4_rev | GCA TTC GCGG CCGC ATC GAT ATC CAT CTC CTC AAC C |
| heat shock 70kDa protein 4 | serotype A / H99 | HSP70_P4_fwd | TC CAA CAT ATG GCC AGT GTC GTC GGT ATT GA |
| hsp71-like protein | serotype A / H99 | HSP71_fwd | TA TTA CAT ATG GTT AAG GCT GTT GGT ATT GAT TTG G |
| hsp71-like protein | serotype A / H99 | HSP71_rev | AAA TGT GCGG CCGC GTC GAC CTC CTC AAC GGA AG |
| hsp71-like protein | serotype D / JEC21 | Hsp71CnIFNTstrepSMx_f | GTT CGA GAA GCC ATG GAT GGT TAA GGC TGT TGG TAT TGA T |
| hsp71-like protein | serotype D / JEC21 | Hsp71CnIFNTstrepSMx_r | ACT GCT GTT ACC ATG GTT AGT CGA CCT CCT CAA CG G |
| hsp72-like protein | serotype A / H99 | HSP72_fwd | TA TTC GGA TCC ATG ACA AAA GCT ATC GGT ATT GAC T |
| hsp72-like protein | serotype A / H99 | HSP72_rev | GCA TTC GCGG CCGC ATC AAC TTC CTC AAC TGA AGG AC |
| hsp75-like protein | serotype A / H99 | HSP75_rev | GCA TTT GCG GCC GCA CGG GCA GAA GCC ATG GC |
| hsp75-like protein | serotype A / H99 | HSP75_fwd | AT TAG CAT ATG TCC GCT GAA GAC GTT TTC GAG |
| hypothetical protein CNAG_05236 | serotype A / H99 | HP_05236_fwd | TA TTA GGA TCC ATG TCT ACA ACG ATG GTC CCA G |
| hypothetical protein CNAG_05236 | serotype A / H99 | HP_05236_rev | GCA TTC GCGG CCGC ATC ATC GTC ACT TTC ACC ATC ACT |
| hypothetical protein CNAG_06113 | serotype A / H99 | HP_06113_fwd | TA TAA CAT ATG TCG GTC GTG TCG AAG AAC CT |
| hypothetical protein CNAG_06113 | serotype A / H99 | HP_06113_rev | TTT AAA GCGG CCGC AGC GCC CAA AGC GGG GAA |
| hypothetical protein CNAG_06946 | serotype A / H99 | HP_06946_fwd | TA GGA CAT ATG CTG CGC ACA GCT TCA AGA AAC |
| hypothetical protein CNAG_06946 | serotype A / H99 | HP_06946_rev | CGA TTA GCGG CCGC CGC CTC AAG TGC CTT CTT TG |
| ketol-acid reductoisomerase | serotype A / H99 | KAD_fwd | TG TTT CAT ATG TCC TTC TCT AGA GCT TCC AGC |
| ketol-acid reductoisomerase | serotype A / H99 | KAD_rev | GCA TTC GCGG CCGC AAG CTC ATC CTT GTT GGC GTC |
| Mannose-1-phosphate guanyltransferase | serotype A / H99 | M1P-G_fwd | TA TGA CAT ATG AAG GCC CTG ATC CTC GTC G |
| Mannose-1-phosphate guanyltransferase | serotype A / H99 | M1P-G_rev | CCA TAC GCGG CCGC CAT AAC AAT ACG GGG CTC AGT G |
| phosphoglucomutase | serotype A / H99 | PGM_fwd | GC TTC CAT ATG TCC AAT ATC ATA ACC GTC AAG ACA |
| phosphoglucomutase | serotype A / H99 | PGM_rev | GCA TTC GCGG CCGC AGT GAT AAC ACT GGG CTT CTC |
| phosphoglucomutase | serotype D / JEC21 | PGM_pET_fwd | CAT CAT CAT AGC GGA TCC ATG TCC GAT ATC GTA ACC GTC A |
| phosphoglucomutase | serotype D / JEC21 | PGM_pET_rev | ATA CAG CTG TGC GGC CGC TTA AGT GAT AAC ACT AGG CTT CTC |
| pyruvate decarboxylase | serotype A / H99 | PyDe_rev | GCA ATT GCGG CCGC GGC CCT GTC GTT GGC TTC |
| pyruvate decarboxylase | serotype A / H99 | PyDe_fwd | TA GGG CAT ATG TCC AGT AAC GAA CAA GTA GCC TT |
| transaldolase | serotype A / H99 | Transald_fwd | CC ACC CAT ATG CCC ACT TCT CTT GAA GCT CTT |
| transaldolase | serotype A / H99 | Transald_rev | GCT TTA GCGG CCGC AGC CTT GAG CTT CTC GAT CAA |
| transketolase | serotype A / H99 | Transketo_fwd | AA TTT CAT ATG GCC AAC TTC TCC AGC AAC GA |
| transketolase | serotype A / H99 | Transketo_rev | CAG CGA GCGG CCGC CTC AGA GAT GTC GTC CAA AGC |
| urease accessory protein UreG | serotype A / H99 | UreG_fwd | TA GTA CAT ATG GCA GTG CCT GCT CAG CCT |
| urease accessory protein UreG | serotype A / H99 | UreG_rev | GCA TTC GCGG CCGC TGC CTT AGC CTT ACC ATT TCC TT |
