# Supplementary material for: Identification of Disease-Associated Cryptococcal Proteins Reactive With Serum IgG From Cryptococcal Meningitis Patients
Source: Front Immunol. 2021 Jul 23;12:709695. doi: 10.3389/fimmu.2021.709695 (PMC8342929; doi:10.3389/fimmu.2021.709695)
Supplement: Supplementary file 8 [file Table_2.docx]

Supplementary Material

# Supplementary Table 2

**Supplementary table 2: Correlation of lung fungal burden with anti-cryptococcal antibody levels in sera from wild type (WT) and IL-4Rα-deficient (IL-4Rα^-/-^) mice.** Correlation analysis was performed using non-parametric Spearman’s rank correlation. Number of colony forming units (CFU) in the lungs of the mice were correlated with titers of anti-protein and anti-CPS IgG and IgM determined by ELISA, or levels of IgG and IgM directed against intact cryptococcal cells (anti-*Cn* IgG, anti-*Cn* IgM). Asterisks indicate significant differences. ns: not significant.

| **Genotype** | **Parameter 1** | **Parameter 2** | **Spearman’s ρ** | **p value** | **Summary** |
| --- | --- | --- | --- | --- | --- |
| WT | Anti-protein IgG | Lung CFU | 0.4017 | 0.0378 | * |
|  | Anti-protein IgM | Lung CFU | 0.1049 | 0.6026 | ns |
|  | Anti-CPS IgG | Lung CFU | 0.4277 | 0.0261 | * |
|  | Anti-CPS IgM | Lung CFU | -0.1579 | 0.4314 | ns |
|  | Anti-Cn IgG | Lung CFU | -0.3755 | 0.0536 | ns |
|  | Anti-Cn IgM | Lung CFU | -0.5364 | 0.0039 | ** |
| IL-4Rα^-/-^ | Anti-protein IgG | Lung CFU | 0.03094 | 0.8537 | ns |
|  | Anti-protein IgM | Lung CFU | 0.1229 | 0.4624 | ns |
|  | Anti-CPS IgG | Lung CFU | -0.04810 | 0.7743 | ns |
|  | Anti-CPS IgM | Lung CFU | -0.04391 | 0.7935 | ns |
|  | Anti-Cn IgG | Lung CFU | 0.04882 | 0.7710 | ns |
|  | Anti-Cn IgM | Lung CFU | 0.06031 | 0.7191 | ns |
