# Supplementary material for: Identification of Disease-Associated Cryptococcal Proteins Reactive With Serum IgG From Cryptococcal Meningitis Patients
Source: Front Immunol. 2021 Jul 23;12:709695. doi: 10.3389/fimmu.2021.709695 (PMC8342929; doi:10.3389/fimmu.2021.709695)
Supplement: Supplementary file 9 [file Table_3.docx]

Supplementary Material

# Supplementary Table 3

**Supplementary table 3: Sub-pools of sera from CM patients and healthy individuals used for 2D immunoproteome analysis.** Quenchable sera from Colombian HIV-positive (HIV+) and HIV-negative (HIV‑) CM patients, as well as healthy control persons were pooled according to their infection status (group) and anti-cryptococcal protein IgG titers (sub-pools) for immunoproteome analysis. CM: cryptococcal meningitis, *Cn*: *Cryptococcus neoformans*. No.: Number.

| **Group** | **Sub-pools** | **Titer anti-*Cn* protein IgG** | **No. of sera** |
| --- | --- | --- | --- |
| HIV+ CM patients | Low titer | 1:100 – 1:200 | 3 |
|  | Intermediate titer | 1: 300 – 1:800 | 4 |
|  | High titer | 1:1600 – 1:12800 | 3 |
| HIV- CM patients | Low titer | 1:800 – 1:3200 | 3 |
|  | Intermediate titer | 1:6400 – 1:12800 | 4 |
|  | High titer | 1:12800 – 1:25600 | 3 |
| Healthy control persons | Low titer | 1:125 – 1:1000 | 4 |
|  | Intermediate titer | 1:1600 | 3 |
|  | High titer | 1:2400 – 1:9600 | 4 |
