# Supplementary material for: Identification of Disease-Associated Cryptococcal Proteins Reactive With Serum IgG From Cryptococcal Meningitis Patients
Source: Front Immunol. 2021 Jul 23;12:709695. doi: 10.3389/fimmu.2021.709695 (PMC8342929; doi:10.3389/fimmu.2021.709695)
Supplement: Supplementary file 13 [file Table_7.docx]

Supplementary Material

# Supplementary Table 7

**Supplementary Table 7: Degree of similarity of the immunoreactive proteins from *C. neoformans* serotype A strain H99 to homologous proteins from the *C. neoformans* serotype D strain JEC21 or *C. gattii* strain WM276 (serotype B).** Comparison of sequence similarity was carried out using the ncbi protein BLAST tool (https://blast.ncbi.nlm.nih.gov/Blast.cgi). Cryptococcus (taxid:5206) was appointed as the organism for search. Acc. No.: Accession number.

| ***C. neoformans* strain H99 serotype A** | | ***C. neoformans* strain JEC21 serotype D** | | | | | | ***C. gattii* strain WM276 serotype B** | | | | | |
| --- | --- | --- | --- | --- | --- | --- | --- | --- | --- | --- | --- | --- | --- |
| **Protein** | **Acc. No.** | **Acc. No.** | **Max score** | **Total score** | **Query Cover** | **E value** | **Percent Identity** | **Acc. No.** | **Max score** | **Total score** | **Query Cover** | **E value** | **Percent Identity** |
| 26S proteasome regulatory subunit N8 | AFR92184 | XP_566467.1 | 714 | 714 | 100% | 0.0 | 99.71% | XP_003191856.1 | 706 | 706 | 100% | 0.0 | 98.29% |
| chlorophyll synthesis pathway protein BchC | AFR97763 | XP_569885.1 | 704 | 704 | 100% | 0.0 | 97.13% | XP_003192587.1 | 709 | 709 | 100% | 0.0 | 97.99% |
| cytoplasmic protein CNAG_02943 | AFR93749 | XP_024514313.1 | 1122 | 1122 | 86% | 0.0 | 98.91% | XP_003193066.1 | 1256 | 1256 | 100% | 0.0 | 97.46% |
| deoxyuridine 5‘-triphosphate nucleotidohydrolase | AFR94562 | XP_572500.1 | 1302 | 1302 | 100% | 0.0 | 95.11% | XP_003196292.1 | 830 | 830 | 58% | 0.0 | 96.58% |
| extracellular elastinolytic metalloproteinase | AFR97484 | XP_567342.1 | 1587 | 1587 | 100% | 0.0 | 94.10% | XP_003196170.1 | 1463 | 1463 | 100% | 0.0 | 85.80% |
| glucose-methanol-choline oxidoreductase | AFR94515 | XP_567934.1 | 1204 | 1204 | 100% | 0.0 | 98.47% | XP_003196508.1 | 1171 | 1171 | 100% | 0.0 | 94.56% |
| glutamate dehydrogenase (NADP) | AFR97782 | XP_569406.1 | 923 | 923 | 100% | 0.0 | 97.34% | XP_003192625.1 | 912 | 912 | 100% | 0.0 | 96.45% |
| glycerol-3-phosphate dehydrogenase (NAD(+)) | AFR92257 | XP_024512024.1 | 685 | 685 | 100% | 0.0 | 95.93% | XP_003191980.1 | 681 | 681 | 100% | 0.0 | 93.90% |
| GTP-binding protein ypt1 | AFR94332 | XP_024513861.1 | 421 | 421 | 100% | 1,00E-151 | 99.51% | XP_003193368.1 | 422 | 422 | 100% | 4,00E-152 | 100.00% |
| heat shock 70kDa protein 4 | AFR98435 | XP_568283.1 | 1555 | 1555 | 100% | 0.0 | 97.67% | XP_003197358.1 | 1500 | 1500 | 100% | 0.0 | 95.99% |
| hsp71-like protein | AFR97929 | XP_569509.1 | 1292 | 1292 | 100% | 0.0 | 98.44% | XP_003192735.1 | 1293 | 1293 | 100% | 0.0 | 98.29% |
| hsp72-like protein, partial | AFR97952 | XP_569545.1 | 1295 | 1295 | 100% | 0.0 | 98.45% | XP_003192750.1 | 1286 | 1286 | 100% | 0.0 | 97.98% |
| hsp75-like protein | AFR92468 | XP_566757.1 | 1256 | 1256 | 100% | 0.0 | 99.67% | XP_003192118.1 | 1255 | 1255 | 100% | 0.0 | 99.51% |
| hypothetical protein CNAG_05236 | AFR94491 | XP_024513811.1 | 937 | 937 | 100% | 0.0 | 99.13% | XP_003196439.1 | 922 | 922 | 100% | 0.0 | 97.84% |
| hypothetical protein CNAG_06113 | AFR98337 | XP_568431.1 | 652 | 652 | 100% | 0.0 | 95.42% | XP_003197250.1 | 468 | 468 | 100% | 1,00E-165 | 94.20% |
| hypothetical protein CNAG_06946 | AFR94883 | XP_570168.2 | 715 | 715 | 100% | 0.0 | 98.85% | XP_003193750.1 | 681 | 681 | 96% | 0.0 | 97.32% |
| ketol-acid reductoisomerase | AFR96043 | XP_571345.1 | 825 | 825 | 100% | 0.0 | 99.50% | XP_003194784.1 | 820 | 820 | 100% | 0.0 | 98.75% |
| Mannose-1-phosphate guanyltransferase | AFR98009 | XP_569600.1 | 742 | 742 | 100% | 0.0 | 99.18% | XP_003195224.1 | 206 | 206 | 98% | 5,00E-62 | 32.32% |
| phosphoglucomutase | AFR98550 | XP_568570.1 | 1131 | 1131 | 100% | 0.0 | 98.22% | XP_003197445.1 | 1098 | 1098 | 100% | 0.0 | 94.47% |
| pyruvate decarboxylase | AFR97558 | XP_567475.2 | 1258 | 1258 | 99% | 0.0 | 97.11% | XP_003193906.1 | 1246 | 1246 | 99% | 0.0 | 96.46% |
| transaldolase | AFR98178 | XP_567910.1 | 640 | 640 | 100% | 0.0 | 95.98% | XP_003196531.1 | 629 | 629 | 100% | 0.0 | 93.50% |
| transketolase | AFR95182 | XP_570402.1 | 1396 | 1396 | 100% | 0.0 | 97.53% | XP_003193474.1 | 1353 | 1353 | 100% | 0.0 | 95.34% |
| urease accessory protein UreG | AFR92807 | XP_566996.1 | 617 | 617 | 100% | 0.0 | 95.24% | XP_003191625.1 | 551 | 551 | 100% | 0.0 | 92.99% |
